# Conjugated Nanohoops Incorporating Donor, Acceptor, Hetero‐ or Polycyclic Aromatics

**DOI:** 10.1002/anie.202007024

**Published:** 2021-03-22

**Authors:** Mathias Hermann, Daniel Wassy, Birgit Esser

**Affiliations:** ^1^ Institute for Organic Chemistry University of Freiburg Albertstr. 21 79104 Freiburg Germany; ^2^ Freiburg Materials Research Center University of Freiburg Stefan-Meier-Str. 21 79104 Freiburg Germany; ^3^ Freiburg Center for Interactive Materials and Bioinspired Technologies University of Freiburg Georges-Köhler-Allee 105 79110 Freiburg Germany

**Keywords:** cyclacenes, cycloparaphenylenes, macrocycles, nanohoops, optoelectronic properties

## Abstract

In the last 13 years several synthetic strategies were developed that provide access to [n]cycloparaphenylenes ([n]CPPs) and related conjugated nanohoops. A number of potential applications emerged, including optoelectronic devices, and their use as templates for carbon nanomaterials and in supramolecular chemistry. To tune the structural or optoelectronic properties of carbon nanohoops beyond the size‐dependent effect known for [n]CPPs, a variety of aromatic rings other than benzene were introduced. In this Review, we provide an overview of the syntheses, properties, and applications of conjugated nanohoops beyond [n]CPPs with intrinsic donor/acceptor structure or such that contain acceptor, donor, heteroaromatic or polycyclic aromatic units within the hoop as well as conjugated nanobelts.

## Graphical abstract

Designer hoops: By incorporating aromatic units with specific electronic (i.e. donor/acceptor) or structural (i.e. chirality) properties, it is possible to modify cycloparaphenylenes and conjugated hoops according to design. This Review provides an overview of such structurally designed hoops, their structural, optoelectronic, and host properties, and potential (future) applications.

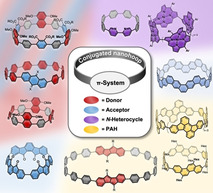

## 1 Introduction

**Figure 1:**
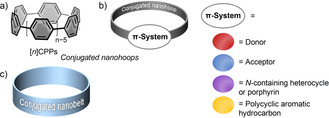
a) [*n*]Cycloparaphenylenes as conjugated nanohoops, b) nanohoops incorporating π‐systems other than benzene (Sections 2–6), and c) nanobelts (Section 7), covered in this review.

Conjugated nanohoops have appealed to chemists for many decades, as they can be used to address fundamental questions, post synthetic challenges, and open up new applications.^[1]^ Their cyclic conjugation, the radial orientation of their π‐system, their rigid structure, size‐dependent physical properties, and host–guest chemistry make them attractive for organic chemists, theoreticians, materials engineers, and physicists.[^2^, ^3^, ^4^, ^5^, ^6^] Bending a π‐system out of planarity can significantly alter its optical, electronic, charge‐transport, and self‐assembly characteristics, giving nanohoops unique properties as light‐emitters, redox‐active molecules, and supramolecular structures. Due to these intriguing properties, conjugated nanohoops were identified as attractive synthetic targets more than 80 years ago.[^5^, ^7^] However, their synthesis remained elusive for decades with only few reported examples of hoops incorporating ring sizes other than six.[^4^, ^8^, ^9^, ^10^] More recent synthetic advances led to the extensive synthesis of nanohoops in the past 12 years, in particular that of [*n*]cycloparaphenylenes ([*n*]CPPs, Figure 1 a),[^11^, ^12^, ^13^] for which synthetic attempts had failed as late as 1994.^[14]^ Amongst the most interesting attributes of [*n*]CPPs are their size‐dependent properties, in particular the invariance of the longest wavelength absorption and the redshift of the emission with decreasing hoop size, which stands in contrast to linear oligo(paraphenylenes).[^12^, ^15^] A number of potential applications emerged for conjugated nanohoops, such as their use as solution‐ and solid‐state fluorophores, as organic electronics components, and as templates for the construction of carbon nanomaterials.[^16^, ^17^, ^18^, ^19^] Furthermore, conjugated nanohoops show rich supramolecular chemistry.[^20^, ^21^] The syntheses[^11^, ^13^] and properties of [*n*]CPPs, including some derivatives,[^22^, ^23^, ^24^] have been reviewed on multiple occasions before,[^12^, ^18^, ^25^, ^26^, ^27^, ^28^] as well as their supramolecular chemistry.[^20^, ^21^] To allow for a tuning of the nanohoop properties beyond the size‐dependent effect, aromatic rings other than benzene were introduced. This Review aims at providing an overview over such nanohoops beyond [*n*]CPPs, that contain not only benzene but other aromatic π‐systems (Figure 1 b). These include nanohoops with intrinsic donor–acceptor structure and hoops incorporating donor, acceptor, heteroaromatic, or polycyclic aromatic units, which will be discussed in Sections 2–6. The color code shown in Figure 1 b will be used to highlight the respective π‐systems within the hoops throughout this Review. Furthermore, conjugated nanobelts will be covered in Section 7 of this Review.

The main incentives to introduce different π‐systems into nanohoops have been to 1) modify their optoelectronic properties, that is, by introducing donor or acceptor moieties or even donor–acceptor structures, 2) modify their structural properties by breaking the high symmetry of [*n*]CPPs leading to chiral nanohoops, and 3) attempt to elongate nanohoops in the vertical direction with the aim to develop methods to synthesize single‐chirality single‐walled carbon nanotubes (SWNTs),[^17^, ^29^] among others. Apart from nanohoop derivatization, synthetic advances have allowed for the synthesis of nanocarbons with unique topologies.[^30^, ^31^]

### 1.1 Synthetic Strategies Leading to Conjugated Nanohoops

**Scheme 1:**
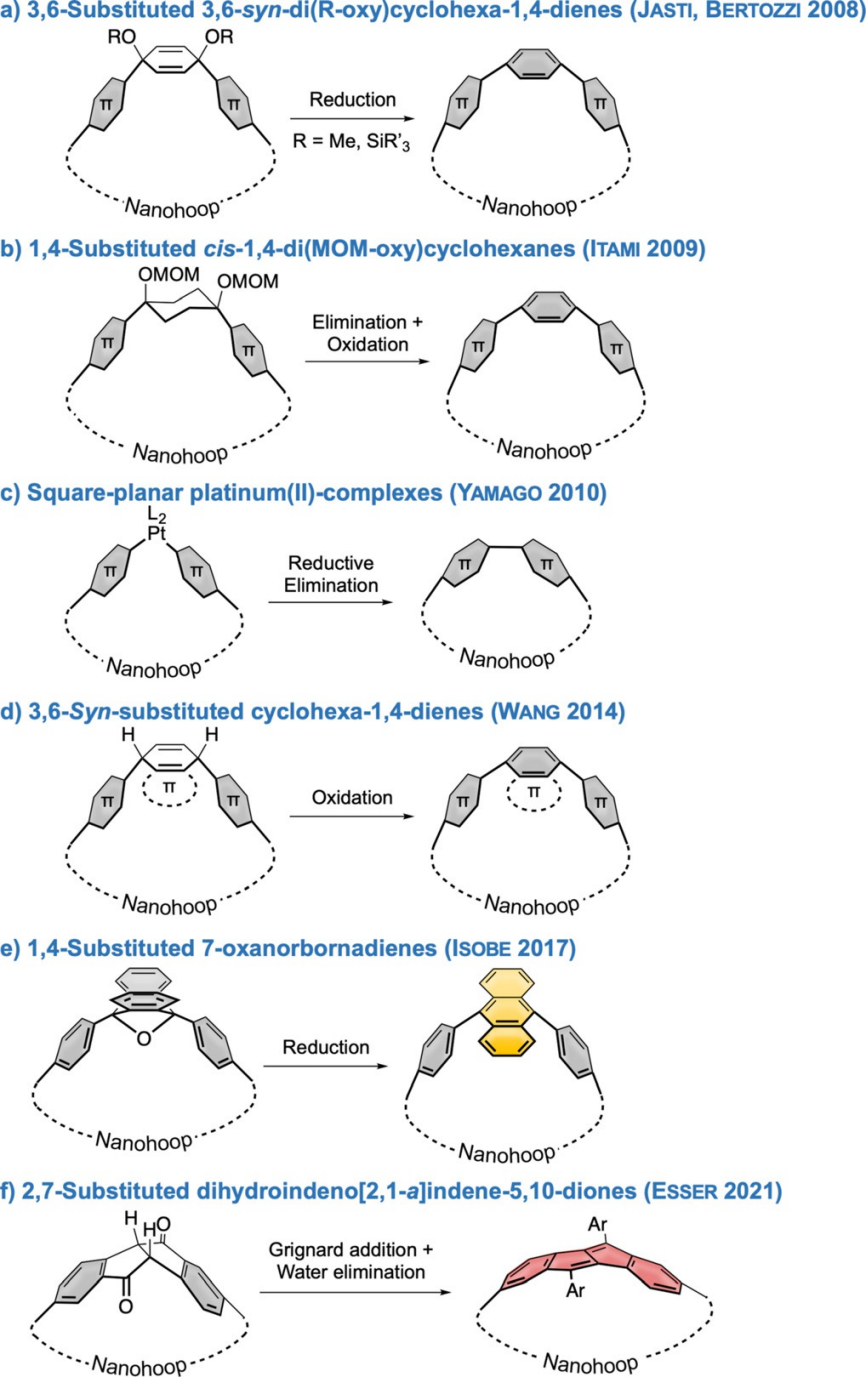
Synthetic strategies to conjugated nanohoops used for the compounds reviewed herein.

The highest challenge in nanohoop synthesis, apart from the macrocyclization step, is to bend the preferably planar π‐system into a hoop shape.^[32]^ The Jasti,^[33]^ Itami,^[34]^ and Yamago^[35]^ groups were the first to develop methods to introduce such a bend in oligo(paraphenylene) units, enabling the synthesis of [*n*]CPPs with *n*=5–16, 18, 20, and 21.[^11^, ^12^, ^13^, ^36^, ^37^] Since then, this synthetic pool has been extended by a number of other methods. The synthetic strategies a)–f) used to access the nanohoops discussed herein are summarized in Scheme 1 and will be referred to throughout this Review. The initial bent precursors developed by Jasti, Bertozzi,^[33]^ (Scheme 1 a) and Itami^[34]^ (Scheme 1 b) can be aromatized by reduction in the first case and elimination + oxidation in the second, while in Yamago's method the aryl–aryl bond is formed by reductive elimination of the square‐planar Pt complex (Scheme 1 c).^[35]^ All three methods have been extensively used in the syntheses of the compounds reviewed herein. In 2014 Wang introduced a cyclohexadiene‐based corner unit (Scheme 1 d), which can be aromatized through oxidation,^[38]^ in the synthesis of naphthalene‐containing nanohoops, discussed in Section 6.2. Isobe employed an oxanorbornadiene derivative as a precursor to a 9,10‐connected anthracene corner unit in 2017(Scheme 1 e),^[39]^ highlighted in the same section. Lastly, our group used a bent and chiral diketone precursor to incorporate dibenzo[*a*,*e*]pentalenes into nanohoops (Scheme 1 f), as will be discussed in Section 6.1.[^40^, ^41^]

### 1.2 Size‐Dependent Properties of [n]Cycloparaphenylenes

[*n*]CPPs possess intriguing size‐dependent properties, which have been reviewed on several occasions.[^12^, ^15^] For comparison with the nanohoops discussed in this Review, we have listed their optical and electrochemical properties in Table 1. Particularly noteworthy is the invariance of the longest wavelength absorption and the redshift of the emission with decreasing hoop size, distinguishing [*n*]CPPs from linear oligoparaphenylenes. As we will highlight, introducing aromatic units other than benzene provides a useful handle to more strongly influence the optoelectronic or structural properties of conjugated nanohoops, for instance through donor or acceptor units (Sections 2–5) or polycyclic aromatic hydrocarbons (Section 6).

**Table 1:** Optoelectronic properties of [*n*]CPPs.

*n* ^[Ref]^	*λ*_max_ [nm]	*ϵ* [m ^−1^ cm^−1^]	*λ*_em_ [nm]	*Φ*_F_	*E*_1/2,Ox_ [V]	*E*_1/2,Red_ [V]
6^[15]^	338	–	–	–	0.44	–
8^[15]^	340^[a]^	1.0×10^5^	533	0.08	0.59	−2.33^[42]^
9^[43]^	339^[a]^	1.3×10^5^	494^[a]^	0.73	0.70^[15]^	–
10^[15]^	341^[a]^	1.3×10^5^	466	0.65	0.74	–
12^[43]^	339^[a]^	1.6×10^5^	426, 450^[a]^	0.89	0.85^[15]^	–
14^[43]^	338^[a]^	–	418, 443^[a]^	0.89	–	–
15^[43]^	339^[a]^	–	416, 440^[a]^	0.90	–	–
16^[43]^	339^[a]^	–	415, 438^[a]^	0.88	–	–
18^[33]^	340	–	412	–	–	–

[a] In CHCl_3_.

## 2 Nanohoops with Donor–Acceptor Structure

**Figure 2:**
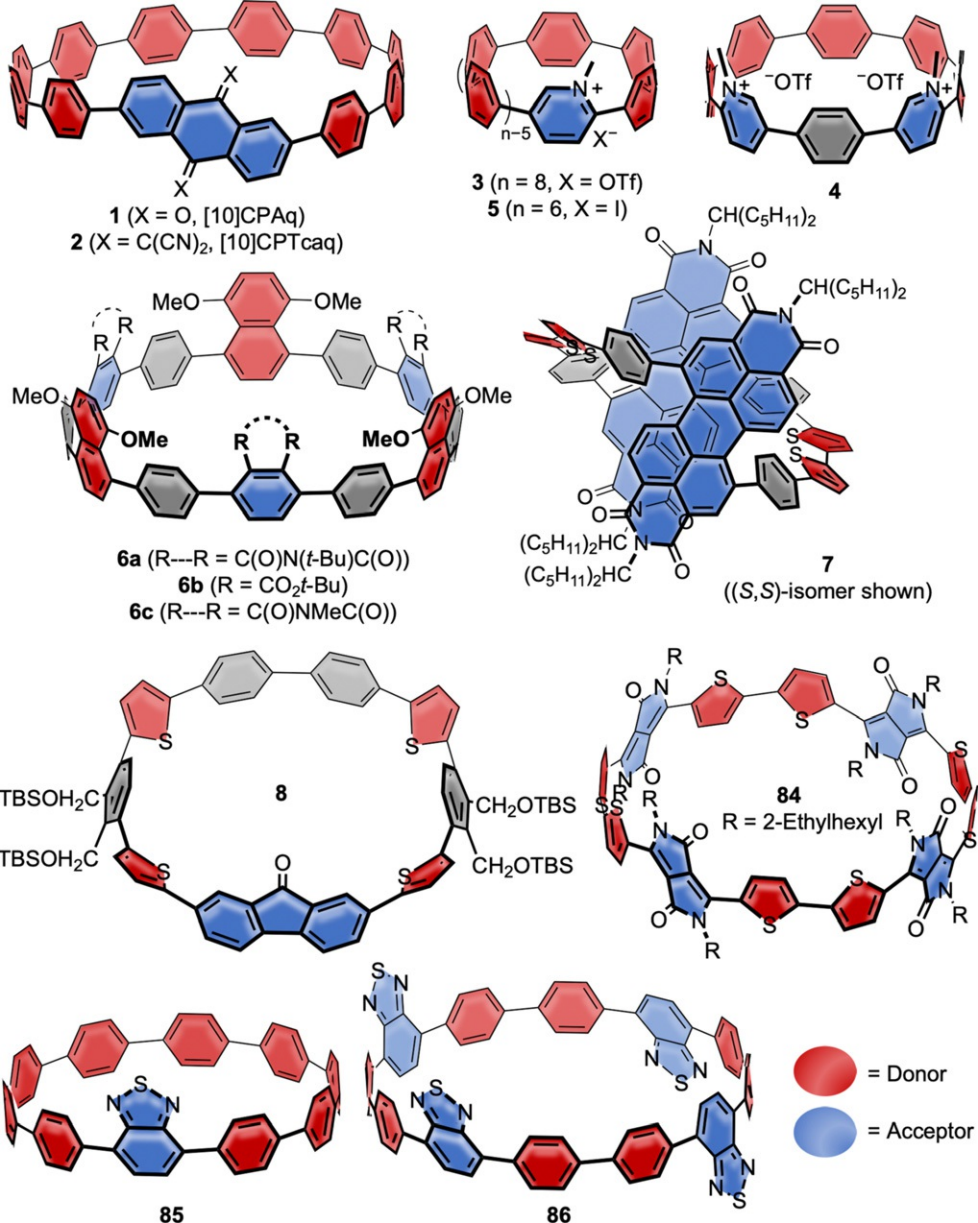
Donor–acceptor nanohoops **1** and **2**,^[45]^ **3** and **4**,^[46]^ **5**,^[47]^ **6 a** and **b**,^[48]^ **7 a/b**,^[49]^ **8**,^[50]^ **84**,^[51]^ **85**,^[52]^ and **86**.^[53]^

Donor–acceptor (D‐A)‐type systems are relevant in many areas, including optoelectronic devices, such as organic solar cells, light‐emitting diodes, and sensors, in addition to biological systems. A D‐A structure typically leads to a lowering of the optical band gap and a spatial localization of the HOMO and LUMO on the donor respective acceptor moiety, among others. Due to the resulting (partial) charge separation in the excited state, a typical observation for D‐A compounds is solvatofluorochromism, where the wavelength of the emitted light depends on solvent polarity,^[44]^ as well as a bathochromically shifted charge‐transfer (CT) band in the absorption spectrum. Introducing a D‐A structure into a nanohoop will significantly alter its optoelectronic properties, as the examples below demonstrate. Nine reports exist to date on nanohoops with intrinsic D‐A structure (see Figure 2 and optoelectronic properties in Table 2). In examples **1**–**5**, **85**, and **86** the donor part is an oligoparaphenylene moiety, while in compounds **6**–**8** and **84** the donor character stems from a dimethoxynaphthalene or from thiophene units. Since CPP subunits have higher HOMO energies than linear oligoparaphenylenes due to their bent π‐system, they can be regarded electronically as donors.

**Table 2:** Optoelectronic properties of donor–acceptor nanohoops discussed in Section 2.

Compound	*λ*_max_ [nm] (solvent)	*ϵ* [m ^−1^ cm^−1^]	*λ*_em_ [nm] (solvent)	*Φ*_F_	*E*_1/2,Ox_ [V]^[a]^	*E*_1/2,Red_ [V]^[a]^	*E*_HOMO_ [eV]^[b]^	*E*_LUMO_ [eV]^[b]^	*E*_gap_ [eV]^[b]^	Ref.
**1** ([10]CPAq)	332 (CHCl_3_)	–	496 (CCl_4_) 523 (C_6_H_6_) 531 (Et_2_O) 591 (C_6_H_5_Cl)	0.08 0.15 0.30 0.18	–	−1.45^[e]^ −1.90^[e]^	−5.39	−2.71	2.68	[45]
**2** ([10]CPTcaq)	335 (CHCl_3_)	–	661 (CCl_4_) 642 (C_6_H_6_)	0.02 0.05	–	−1.85^[f]^	−5.48	−3.55	1.93	[45]
**3**	345 (CH_2_Cl_2_)	2.9×10^4^	598 (CH_2_Cl_2_)	–	–	−1.49^[g,h]^	−5.36	−2.92	2.44	[46]
**4**	350 (CH_2_Cl_2_)	4.9×10^4^	630 (CH_2_Cl_2_)	—	–	−1.36,^[g,h]^ −1.49^[g,h]^	−5.63	−3.07	2.56	[46]
**5**	343 (CH_2_Cl_2_)	2.3×10^4^	–	–	0.66^[c,i]^	−1.49,^[g,i]^ −2.47,^[g,i]^ −2.72^[g,i]^	−5.39	−2.68	2.71	[47]
**6 a**	339, 354 (CHCl_3_)	–	481 (^[c]^) 549 (CHCl_3_)	0.23 0.021	–	–	−4.95	−2.05	2.90	[48]
**6 b**	300, 350 (CHCl_3_)	–	475 (^[c]^) 484 (CHCl_3_) 497 (^[d]^)	0.48 0.59 0.43	—	–	−4.88	−1.42	3.46	[48]
**7**	300–400 (CH_2_Cl_2_) *E*_gap‐opt_=1.80 eV	–	–	–	0.58^[e or f,i]^	−1.07^[e or f,i]^	–	–	–	[49]
**8**	340 (CH_2_Cl_2_)	2.3×10^4^	454, 573 (CH_2_Cl_2_)	–	0.82,^[g]^ 0.92^[g]^	−1.65^[f]^	–	–	–	[50]
**84**	570 (−) 600 (thin film)	–	700–1000 (thin film)	–	0.58^[g,j]^	−1.56^[g,h]^	−4.80	−3.19	1.61	[51]
**85** (BT[10]CPP)	334 (CH_2_Cl_2_)	5.4×10^4^	571 (CH_2_Cl_2_)	0.59	0.78^[e]^	–	−6.53^[k]^	−1.92^[k]^	4.61^[k]^	[52]
**86** (TB[12]CPP)	320 (CHCl_3_)	8.3×10^4^	569 (CHCl_3_) 555 (CCl_4_) 586 (DMF)	0.81 0.82 0.59	–	–	–	–	–	[53]

[a] From cyclic voltammetry vs. Fc/Fc^+^. [b] B3LYP/6‐31G*. [c] In *n*‐hexane, 5 % CHCl_3_. [d] In MeCN, 5 % CHCl_3_. [e] Reversible. [f] Quasi‐reversible. [g] Irreversible. [h] Cathodic peak potential. [i] Onset reduction or oxidation peak. [j] Anodic peak potential. [k] CAM‐B3LYP/6‐31G* with CH_2_Cl_2_ as solvent.

**Figure 3:**
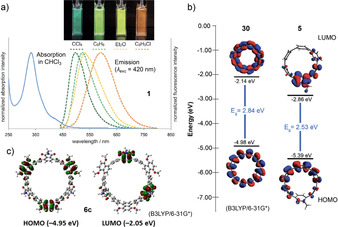
a) Optical spectra of **1** with photographs of emission colors, reprinted with permission from ref. [45]; copyright 2015 Wiley‐VCH. b) Frontier molecular orbitals of **30** and methylated **5**, reproduced from ref. [47] with permission from The Royal Society of Chemistry. c) Frontier molecular orbitals of **6 c**, reprinted with permission from ref. [48]; copyright 2017 Wiley‐VCH.

The first D‐A nanohoop (**1**) was reported by Itami's group in 2015, who introduced an anthraquinone moiety as an acceptor into a [10]CPP donor.^[45]^ Suzuki–Miyaura coupling reactions were used to connect a 2,6‐disubstituted anthraquinone with an “Itami corner unit” (Scheme 1 b), and a Ni‐mediated Yamamoto coupling was employed to perform the ring closure. The acceptor character was further increased by transforming the anthraquinone moiety into a tetracyanoanthraquinodimethane group (**2**). While the absorption maxima of **1** (332 nm) and **2** (335 nm) were almost identical to that of parent [12]CPP (338 nm), the fluorescence differed. **1** exhibited green fluorescence in CCl_4_ (*λ*_max‐em_=496 nm) with a bathochromic shift to orange (591 nm) in the more polar chlorobenzene (Figure 3 a). **2** showed red fluorescence in CCl_4_ and benzene with a bathochromic shift, but was non‐emissive in more polar solvents. Parent [12]CPP, for comparison, exhibits blue fluorescence. Calculations localized the electron density of the HOMOs on the oligoparaphenylene moieties and that of the LUMOs on the acceptor units with similar HOMO energies of −5.39 eV for **1**, −5.48 eV for **2**, and −5.25 eV for [12]CPP, while the LUMO energies were strongly affected with −2.71 eV for **1**, −3.55 eV for **2**, and −1.64 eV for [12]CPP in comparison.

Also in 2015, the Jasti group published another approach to D‐A nanohoops by introducing pyridinium moieties as acceptors (**3** and **4**) into CPPs as donors.^[46]^ The syntheses were performed using a Suzuki–Miyaura coupling in the macrocyclization step and employed the Jasti corner unit (Scheme 1 a). Several aza[8]CPPs with one, two, or three pyridine units replacing phenylene rings were synthesized (**27**–**29** in Figure 8, see also Section 5.1). The electronic properties of these aza[8]CPP were quite similar to [8]CPP in spite of the presence of the pyridine rings, hence they will be discussed in Section 5.1. In the corresponding methylated hoops **3** and **4**, on the other hand, obtained by reaction with methyl triflate, the acceptor character of the pyridine unit was strengthened, resulting in oxidation potentials shifted by ca. 1 V towards higher voltages. DFT calculations confirmed the experimental data with a lowering of the LUMO energy by 1.15 eV for **4** relative to [8]CPP. The D‐A hoops **3** and **4** showed a bathochromic shift in the absorption maxima compared to [8]CPP with a broad shoulder between 400 and 425 nm. The fluorescence significantly redshifted to 598 nm (**3**) and 630 nm (**4**) compared to [8]CPP and the non‐methylated aza[8]CPPs **27**–**29**. The Jasti group further followed up with the smaller derivative aza[6]CPP (**30** in Figure 8, see Section 5.1) and *N*‐methylaza[6]CPP (**5**).^[47]^ Their recently developed Pd‐catalyzed oxidative homocoupling of boronic esters, which was successful in the synthesis of highly strained [5]CPP,^[48]^ was employed for ring‐closing.^[54]^ Similar to aza[8]CPP, the smaller aza[6]CPP **30** showed little change in optoelectronic properties compared to [6]CPP. After methylation to **5**, on the other hand, the reduction potential was shifted upwards by 0.71 V (to −1.42 V) compared to aza[6]CPP **30**. The HOMO and LUMO of both compounds further confirmed the electronic nature of a D‐A system (Figure 3 b). This strengthened the previous findings of aza[8]CPP and its methylated derivative, that simple *N*‐substitution has little effect on the energy levels and only methylated aza[*n*]CPPs possess D‐A character.

The Tanaka group employed a Rh‐catalyzed cross‐cyclotrimerization of a diyne to obtain D‐A‐[12]CPPs with alternating 1,4‐dimethoxynaphtalene units as donor and phthalimide or phthalate esters as acceptor separated by *p*‐phenylene units (**6 a** and **b**).^[48]^ A 1,4‐dimethoxy‐5,8‐dihydronaphthalene unit acted as the bent aromatic precursor (Scheme 1 d) with yields up to 13 % over two steps (cyclization and aromatization). Single‐crystal X‐ray diffraction confirmed the conformation shown in Figure 2 as all*‐syn*, in which the acceptor and donor moieties faced each other despite steric bulkiness. The molecules of **6 a** adopted a columnar packing structure with CH–π interactions between a *t*‐butyl hydrogen atom and the dimethoxynaphthalene moieties. Both **6 a** and **b** showed a bathochromic shift in the absorption spectra and a strong positive solvatofluorochromism due to their D‐A structure. This was also seen in the HOMO and LUMO electron density distribution calculated for **6 c** (Figure 3 c).

In 2015 Ball et al. published their work on bithiophene–perylene diimide (PDI) donor–acceptor hoop **7**, called “conjugated corral” due to its shape.^[49]^ By using stannylated precursors and a Pt‐mediated coupling reaction (Yamago method, Scheme 1 c), the authors were able to obtain all three stereoisomers (meso compound (*S*,*R*) and enantiomers (*S*,*S*) and (*R*,*R*)) via separation by chiral HPLC. The enantiomers interconverted at room temperature via the meso isomer, reaching equilibrium after two hours. The optoelectronic properties reflected the presence of both the bithiophene and the PDI units in addition to a new bathochromically shifted band in the absorption spectrum.

A similar concept—using donors and acceptors frequently employed in co‐polymers for organic electronics applications—was reported by Li and co‐workers in 2019, who synthesized nanohoop **84** with alternating diketopyrrolopyrrol (DPP) and bithiophene units.^[51]^ Yamago's Pt‐mediated route (Scheme 1 c) was employed, and a linear reference compound with the same number of DPP and thiophene units served for comparison. DFT calculations showed **84** to possess nearly circular shape. Broad photoluminescence in the range of 700–1000 nm was observed. The potential applicability of D‐A‐nanohoop **84** was demonstrated in three different device measurements: A simple organic light‐emitting diode measurement showed electroluminescence at 800–1000 nm with an external quantum efficiency of about 0.0001 %; an organic field‐effect transistor measurement provided ambipolar charge transport; and when **84** was used as non‐fullerene acceptor with P3HT as the donor in a bulk heterojunction (BHJ) solar cell, an initial power conversion efficiency of 0.49 % was found.

In 2017 the Wang group incorporated a fluorenone unit as an acceptor into a thiophene‐ and phenylene‐containing hoop (**8**).^[50]^ They performed the ring closure using a Ni‐mediated Yamamoto coupling and employed an oxidative aromatization of a cyclohexadiene precursor (Scheme 1 d). **8** can be compared with [10]CPP by replacing four benzene rings with thiophenes and two benzene rings with the fluorenone group. **8** exhibited an intramolecular CT band in the absorption spectrum and solvatofluorochromic behavior with a bathochromic shift for solvents of higher polarity (545 nm in cyclohexane to 597 nm in CHCl_3_). In contrast to [10]CPP, **8** showed two emission bands in CH_2_Cl_2_, with one at 573 nm significantly redshifted. In the CV a reduction event was visible, which can be attributed to the fluorenone group, while the oxidation wave was shifted towards higher potential compared to [10]CPP. Two larger rings with two and three fluorenone groups were also obtained using a thiophene–thiophene Yamamoto coupling.

In 2020 the Jasti group published the benzothiadiazole‐containing [10]CPP derivative **85** (BT[10]CPP).^[52]^ Synthesis was afforded by Suzuki–Miyaura coupling of dibromobenzothiadiazole with a C‐shaped synthon containing three Jasti corner units (Scheme 1 a) followed by reductive aromatization. While with 334 nm the absorption maximum of **85** was almost unchanged compared to [10]CPP (341 nm), the emission maximum was redshifted by 105 nm to 571 nm. Remarkably, the fluorescence quantum yield (FQY) remained high (*Φ*_F_=0.59) compared to [10]CPP (*Φ*_F_=0.65), which stands in contrast to other D‐A nanohoops (see Table 2) and made this the first bright orange‐emitting nanohoop. An even brighter orange emission was obtained for benzothiadiazole‐containing nanohoop **86** (TB[12]CPP), published shortly afterwards in 2020. The Tan group synthesized **86** using Yamago's Pt‐mediated route and confirmed its spherical structure by single‐crystal X‐ray diffraction.^[53]^ **86** possessed two absorption maxima at 320 and 428 nm, with the latter being redshifted by 89 nm compared to parent [12]CPP. With 569 nm the emission maximum was even more redshifted compared to [12]CPP (450 nm), and **86** showed positive solvatofluorochromism due to its D‐A character. The FQY reached new record values as high as *Φ*_F_=0.82 in solution for orange‐emitting nanohoops. In addition, the supramolecular chemistry of **86** was explored using anthracene‐C_60_, among others, as a guest molecule.

## 3 Acceptor‐Containing Nanohoops

**Figure 4:**
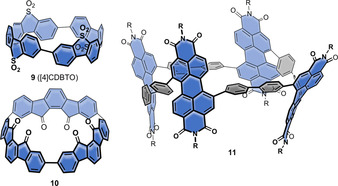
Acceptor‐containing nanohoops **9**,^[42]^ **10**,^[55]^ and **11**.^[56]^

Introducing electron acceptors into nanohoops lowers their orbital energies. This is particularly interesting for application in optoelectronic devices, if good n‐type conduction is required and where a LUMO energy below −3 eV is desired. Three reports exist on such nanohoops, shown in Figure 4, and with optoelectronic properties listed in Table 3.

**Table 3:** Optoelectronic properties of acceptor‐ and donor‐containing nanohoops discussed in Sections 3 and 4.

Compound	*λ*_max_ [nm] (solvent)	*ϵ* [m ^−1^ cm^−1^]	*λ*_em_ [nm] (solvent)	*Φ*_F_	*E*_1/2,Ox_ [V]^[a]^	*E*_1/2,Red_ [V]^[a]^	*E*_HOMO_ [eV]^[b]^	*E*_LUMO_ [eV]^[b]^	*E*_gap_ [eV]^[b]^	Ref.
**9**	337 (CHCl_3_)	–	429, 529 (CHCl_3_)	0.41	–	−1.57^[d]^	−6.01	−2.92	3.09	[42]
**10**	288 (CH_2_Cl_2_)	9.2×10^3^	448, 606 (CH_2_Cl_2_)	–	–	−1.47^[c]^	–	–	–	[55]
**11**	ca. 600 *E*_gap‐opt_ =2.12 eV	–	–	–	*E*_HOMO_=−5.39 eV^[h]^	*E*_LUMO_=−3.90 eV^[h]^	–	–	–	[56]
**[4]12** ([4]CPT)	333 (C_6_H_12_)	–	546 (C_6_H_12_)	–	–	–	−5.01	−1.74	3.27	[59]
**[5]12** ([5]CPT)	350 (C_6_H_12_)	–	510 (C_6_H_12_)	–	–	–	−5.00	−1.82	3.18	[59]
**[6]12** ([6]CPT)	362 (C_6_H_12_)	–	488 (C_6_H_12_)	–	–	–	−4.97	−1.90	3.07	[59]
**[2]13**	363 (CH_2_Cl_2_)	–	512 (CH_2_Cl_2_)	–	0.41,^[d]^ 0.79^[e]^	–	–	–	–	[60]
**[4]13**	350 (CH_2_Cl_2_)	–	481 (CH_2_Cl_2_)	–	0.53,^[d]^ 0.84,^[d]^ 0.98^[d]^	–	–	–	–	[60]
**14**	376 (CH_2_Cl_2_)	–	511 (CH_2_Cl_2_)	–	0.49,^[d]^ 0.93^[d]^	–	–	–	–	[60]
**15** ([4]CDBT)	337 (CHCl_3_)	–	510 (CHCl_3_)	0.21	0.74,^[c]^ 0.96^[c]^	−2.07^[d]^	−5.23	−1.88	3.35	[42]
**16**	363 (CH_2_Cl_2_)	–	481 (CH_2_Cl_2_)		0.37,^[d]^ 0.81,^[d]^ 1.02^[e]^	–	–	–	–	[61]
**[2]17** (R=TIPS)	365 (CH_2_Cl_2_)	–	502 (CH_2_Cl_2_)	–	0.28,^[d]^ 0.67,^[e]^ 0.91,^[e]^ 1.17^[e]^	–	–	–	–	[61]
**[3]17** (R=TIPS)	382 (CH_2_Cl_2_)	–	486 (CH_2_Cl_2_)	–	0.37,^[d]^ 0.81,^[d]^ 1.02,^[e]^ 1.21^[e]^	–	–	–	–	[61]
**18**	266, 363 (CH_2_Cl_2_)	2.7×10^4^, 2.6×10^4^	465 (CH_2_Cl_2_)	0.18	0.057^[d]^, 0.18^[d]^	–	−5.01	−2.39	2.62	[62]
**19**	319 (CH_2_Cl_2_)	–	431 (CH_2_Cl_2_)	0.06	0.12^[e,h]^	–	−5.15	−2.18	2.97	[62]
**20 a**	263, 338 (CHCl_3_)	5.0×10^4^, 4.5×10^4^	486 (CHCl_3_)	0.18	0.28^[c]^	–	−4.89	−1.36	3.53	[63]
**20 b**	262, 341 (CHCl_3_)	4.6×10^4^, 3.2×10^4^	488 (CHCl_3_)	0.18	0.32^[c]^	–	−4.87	−1.31	3.56	[63]
**20 c**	262, 340 (CHCl_3_)	8.4×10^4^, 7.7×10^4^	494 (CHCl_3_)	0.14	0.40,^[e,g]^ 0.94,^[e,g]^ 1.57^[e,g]^	−2.90,^[e,f]^ −3.01^[e,f]^	–	–	–	[64]
**21**	–	–	–	–	–	–	−4.87	−1.4	3.47	[63]
**22**	357 (CH_2_Cl_2_)	1.4×10^5^	496 (CH_2_Cl_2_)	0.36	0.64^[e]^	−2.36^[e]^	−5.10	−1.75	3.35	[65]

[a] From cyclic voltammetry vs. Fc/Fc^+^.^[66]^ [b] B3LYP/6‐31G*. [c] Reversible. [d] Quasi‐reversible. [e] Irreversible. [f] Cathodic peak potential. [g] Anodic peak potential. [h] Onset reduction or oxidation peak.

The Yamago group expanded the scope of their Pt‐mediated cyclotetramerization (Scheme 1 c) from using biphenyl groups to dibenzothiophenes to furnish sulfone‐substituted [8]CPP derivative **9** (see Figure 4, synthesis via **15** in Section 4.1) in 2017.^[42]^ With the help of NMR spectroscopy, DFT calculations, and single‐crystal X‐ray diffraction, they confirmed the *anti*/*anti*/*anti* conformation of **9** to be thermodynamically most favored and 9.8 kcal mol^−1^ lower in energy than the *syn*/*syn*/*syn* conformation. The absorption maximum of 337 nm was nearly identical to that of parent [8]CPP (340 nm). The fluorescence emission wavelength was also similar to [8]CPP, but the FQY much higher with *Φ*_F_=0.41 compared to 0.081 ([8]CPP). The more rigid structure of the sulfur‐containing hoop was probably the reason for the higher quantum yield. Cyclic voltammetry of **9** showed no oxidation but a quasi‐reversible reduction wave at −1.57 V vs. Fc/Fc^+^, which is shifted to less negative values compared to [8]CPP with −2.33 V.

In 2017 the Wang group used their hexaester‐containing [9]CPP^[57]^ in a Friedel–Crafts acylation to obtain nanohoop **10**, consisting of three indenofluorenedione groups and bearing six carbonyl groups in total.^[55]^ NMR studies and DFT calculations revealed the *anti*/*syn* conformation (shown in Figure 4) as the most stable, 4.3 kcal mol^−1^ lower in energy than the all‐*syn* isomer and with a rotational barrier of Δ*G* ^≠^=23.3 kcal mol^−1^ for one indenofluorenedione group. The optoelectronic properties were dominated by the indenofluorenedione moieties rather than the parent [9]CPP structure. The absorption maximum of **10** at 288 nm was hypsochromically shifted compared to [9]CPP (340 nm), and two fluorescence maxima appeared at 448 and 606 nm upon excitation at 395 nm. The cyclic voltammogram showed a reversible reduction with a half‐wave potential of −1.47 V vs. Fc/Fc^+^, whereas parent [9]CPP has no observable reduction wave in the respective electrochemical window.

Ball et al. expanded their work on “conjugated corrals” with the synthesis of **11**, containing four PDI units connected via biphenyl units, which was applied as an n‐type material in organic electronics devices.^[56]^ **11** as well as **7 a/b** (Figure 2) were compared to their respective linear analogues, monomeric units, and polymers. Organic photovoltaic devices were fabricated with each compound used as acceptor in a BHJ cell. The cyclic compounds performed better than the acyclic ones since they absorbed more visible light and showed a better energy alignment with the donor material, higher electron transport mobilities, and an optimal phase separation for BHJ solar cells. The absorption of **11** was bathochromically shifted compared to the acyclic molecules, indicative of a smaller HOMO–LUMO gap. **11** was also later used in an organic photodetector.^[58]^

## 4 Donor‐Containing Nanohoops

**Figure 5:**
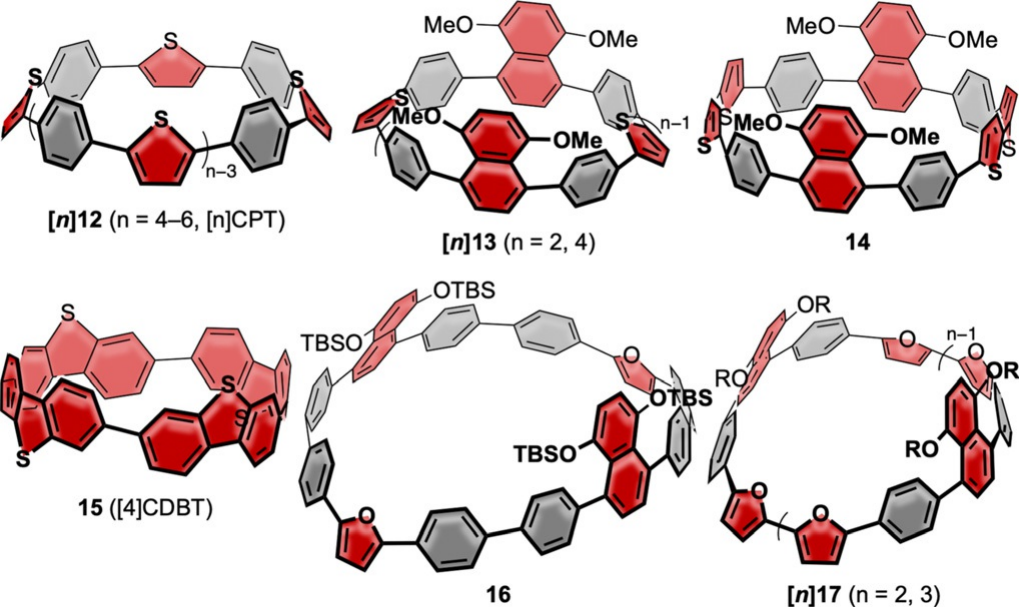
Thiophene‐ and furan‐containing nanohoops **[*n*]12**,^[59]^ **[*n*]13** and **14**,^[60]^ **15**,^[42]^ and **16** and **[*n*]17**.^[61]^

In contrast to the acceptors discussed above, introducing a donor moiety can increase orbital energies. In addition, introducing S‐containing heterocycles, such as thiophenes, will impact molecular packing in the solid state due to their strong van der Waals interactions.^[67]^ Thiophenes, furans, and carbazoles as donor moieties were introduced into nanohoops, as discussed in this section (see Figure 5 and Table 3). This Review excludes cyclo[*n*]thiophenes,^[68]^ solely consisting of thiophene units, since they do not feature radial π‐conjugation due to the bond angles of the thiophene moiety.

### 4.1 Thiophene and Furan

**Figure 6:**
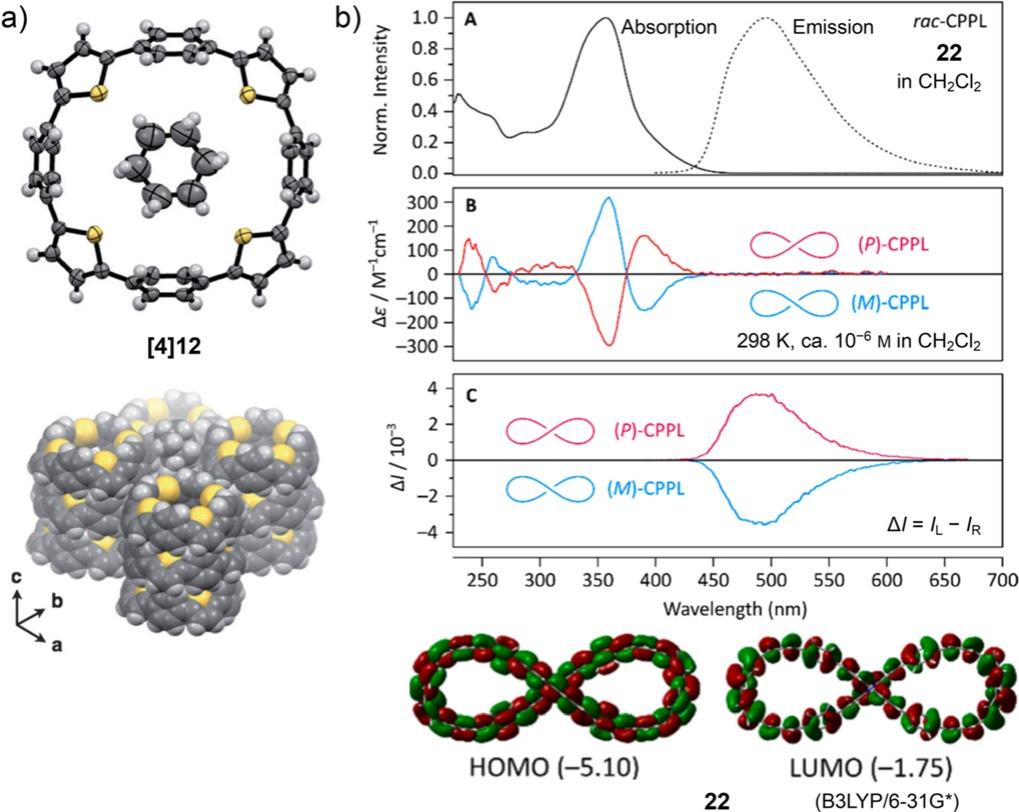
a) Molecular structure of **[4]12** in the solid state (thermal ellipsoids shown at 50 % probability level, with enclosed cyclohexane) and tubular packing structure, reprinted with permission from ref. [59]; copyright 2015 Wiley‐VCH. b) Optical spectra of **22** (A), electronic circular dichroism spectra (B), and circularly polarized luminescence spectra (C) of the (*P*) and (*M*) isomers, and frontier molecular orbitals, reprinted with permission from ref. [65]; copyright 2019 American Chemical Society.

The Itami group synthesized a series of alternating *para*‐phenylene‐ and thiophenylene‐containing nanohoops consisting of 4, 5, and 6 units each ([*n*]CPT, **[*n*]12** in Figure 5) in 2015.^[59]^ The synthesis was performed using their “Itami corner” (Scheme 1 b) with two alkyne units, which were coupled using a Glaser–Hay cyclo‐oligomerization. This approach yielded cyclic diynes of different sizes ranging from dimers to hexamers, separable by column chromatography, which were transformed into the thiophenes using Na_2_S. After aromatization of the cyclohexane moieties, only hoop sizes *n*=4–6 could be obtained because of the low thermal stability of the higher strained **[3]12**. The structure of **[4]12** was confirmed by X‐ray diffraction and showed the molecule with the thiophene units all pointing in the same direction (Figure 6 a). The molecules adopted a tubular structure, in contrast to [*n*]CPPs, which usually pack in a herringbone structure. **[*n*]12** showed a slight bathochromic shift in the absorption spectra compared to the respective [*n*]CPP with increasing hoop size (333 nm for *n*=4 to 362 nm for *n*=6). Like [*n*]CPP, the fluorescence emission showed a blueshift with increasing ring size.^[69]^

The Wang group synthesized a series of dimethoxynaphthalene‐ and thiophene‐containing nanohoops in 2015.^[60]^ In their synthesis they used a dihydronaphthalene corner unit (Scheme 1 d), and the cyclization was performed using a Suzuki–Miyaura coupling towards **[*n*]13** and a Ni‐mediated dimerization towards **14**. The most stable conformer of **[2]13** was the *syn* isomer with both naphthalene und sulfur atoms pointing in the same direction. VT‐NMR measurements revealed a rotational barrier of the *p*‐phenylene groups of Δ*G* ^≠^=11 kcal mol^−1^ at a coalescence temperature of −41 °C. For **14** the *anti* isomer was the most stable with the naphthalene groups pointing in a different direction than the thiophene sulfur atoms, while for large hoop **[4]13** it was the *syn* conformation. The fluorescence maxima were blueshifted for the larger **[4]13** compared to **[2]13**, as known for [*n*]CPPs, but contrary to the previously mentioned **[*n*]12**.

As mentioned in Section 3, the Yamago group synthesized the cyclic tetramer of dibenzothiophene **15** ([4]CDBT) as the [8]CPP derivative in 2017.^[42]^ The *anti*/*anti*/*anti* structure was the thermodynamically most favored and 1.7 kcal mol^−1^ lower in energy than the *syn*/*syn*/*syn* conformation. With 337 and 510 nm the respective absorption and fluorescence maxima were close to the values for [8]CPP (340 nm and 533 nm, respectively). Yet the FQY (*Φ*_F_=0.21) was higher than for [8]CPP (*Φ*_F_=0.08), likely due to the more rigid structure of **15**. Cyclic voltammetry of **15** gave two reversible oxidations at 0.74 and 0.96 V vs. Fc/Fc^+^ which was higher than that of [8]CPP with 0.59 V. Calculated HOMO/LUMO energy levels revealed the same behavior regarding the HOMO–LUMO energy gap for cyclic vs. linear structures. Cyclic **15** had a HOMO–LUMO energy gap of 3.35 eV, its linear analogue [4]DBT 3.78 eV.

The Wang group investigated furan derivatives **16** and **[*n*]17**, analogues of their thiophene‐containing nanohoops, in 2016.^[61]^ The synthesis was similar to that of their sulfur analogues using Suzuki and Yamamoto couplings with dihydronaphthalenes as aromatic precursors (Scheme 1 d). They obtained the [12]CPP derivative **16** with two furan and two dialkoxynaphthalenes and the [10]‐ and [15]CPP derivatives **[*n*]17** with four (*n*=2) and six (*n*=3) furans, and two and three naphthalenes, respectively. The structure of **[2]17** (R=TIPS) was confirmed by X‐ray diffraction and showed a conformation in which all furan oxygen atoms and naphthalene units pointed in the same direction. This stands in contrast to thiophene‐based **14**, where the thiophene units pointed in the other direction.^[60]^ Interestingly the aromatization reaction of both precursors with different relative configuration of the naphthalene groups (*anti* and *syn*) yielded **[2]17** in the *anti* conformation in similar yields. The optical properties were similar to **[*n*]12** and [*n*]CPPs. There was a redshift in the absorption (365 to 382 nm) and emission (502 to 486 nm) maxima with increasing hoop size from **[2]17** to **[3]17. [2]17** had similar properties as **14** with a slight blueshift (11 nm) of the absorption maximum and a similar emission wavelength. The electrochemical properties followed the trend for [*n*]CPPs with lower oxidation potentials with decreasing hoop size, as had been the case for the thiophene‐containing compound. The half‐wave potentials were lower for the furan than for the thiophene derivatives, that is, 0.21 V lower for **[2]17** (0.28 V vs. Fc/Fc^+^) compared to **14**. This is even lower than in [10]CPP with a value of 0.74 V vs. Fc/Fc^+^.

### 4.2 Carbazole

**Figure 7:**
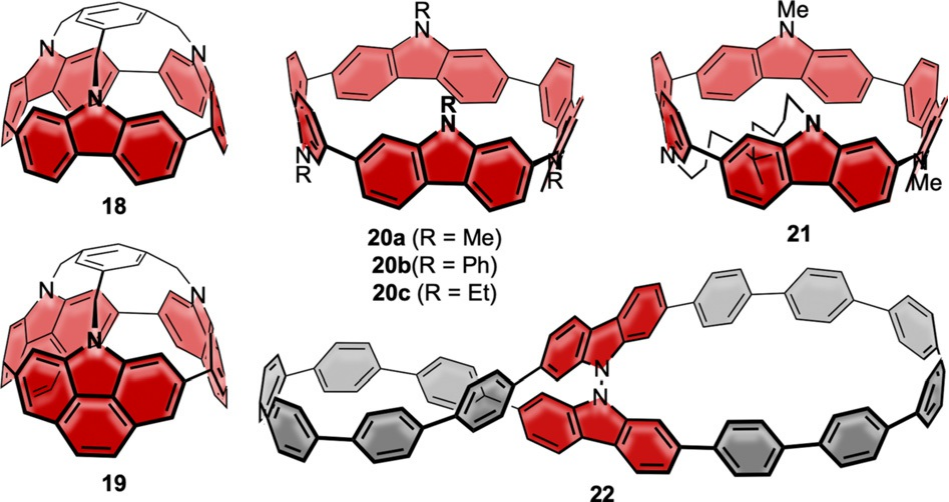
Carbazole‐containing nanohoops **18** and **19**,^[62]^ **20 a** and **b** and **21**,^[63]^ **20 c**,^[64]^ and **22**.^[65]^

Carbazole is an electron‐rich heterocycle extensively used in molecules for optoelectronics. This is due to its p‐type character and the good hole mobility of its derivatives, and it was lately used as the donor in OLED emitters with D‐A structure, showing thermally activated delayed fluorescence.^[70]^ Carbazole was incorporated into nanohoops in three different types of architectures, shown in Figure 7. Their optoelectronic properties are listed in Table 3.

Stępień and co‐workers synthesized two capped [3]cyclocarbazole derivatives in 2015, **18** and **19**.^[62]^ They used a mesitylene derivative as the cap and internal template to facilitate ring closure, which they performed using a Ni‐mediated Yamamoto coupling. **18** and **19** can be regarded as derivatives of [6]CPP, **18** with three 2,7‐carbazole groups and **19** with three 2,7‐benzo[*def*]carbazole groups. The ring strain, however, was significantly higher than that of [6]CPP (98 kcal mol^−1^) with a value of up to 144 kcal mol^−1^, which lies in the region of [4]CPP.^[71]^ Possible reasons are the limited conformational freedom with H–H repulsion, steric congestion because of the capping, and the increased rigidity of the carbazole units. Both structures were confirmed by X‐ray diffraction. The absorption spectra of **18** and **19** were quite different from that of [6]CPP. **18** showed two main absorptions of similar intensity and, unlike non‐emissive [6]CPP, was blue‐fluorescent with maxima at 440 nm and 465 nm. These bands likely corresponded to transitions from a higher excited state than S_1_. In the CV quasi‐reversible oxidations of **18** occurred at 0.06 and 0.18 V vs. Fc/Fc^+^, which lie 0.24 V lower than for [6]CPP, demonstrating the influence of the electron‐rich carbazole units.

In 2016, the Suzuki group synthesized a series of [4]cyclocarbazoles **20 a** and **b** and **21**, as derivatives of [8]CPP, using the Pt‐mediated cyclization approach (Scheme 1 c) from stannylated precursors.^[63]^ **21** has a bridging alkyl linker, which was initially meant to exert a template effect. They confirmed the all‐*anti* conformer to be the most stable by DFT calculations as well as by X‐ray diffraction of **20 a**. Like for the smaller **18**, two main absorption maxima were observed, with the lower wavelength absorption in the region of [*n*]CPPs (≈340 nm). The fluorescence maximum was observed at 486 nm for **20 a** with an FQY of *Φ*_F_=0.18, 47 nm blueshifted compared to [8]CPP and with more than double the FQY. In the CV the carbazole derivatives showed a more facile and reversible oxidation than [8]CPP, with voltages below 0.31 V vs. Fc/Fc^+^.

The Poriel group further investigated ethyl‐substituted [4]cyclocarbazole **20 c** in 2019 to gain more insight into its photophysical properties.^[64]^ They optimized the Pt‐mediated cyclization (Scheme 1 c) using borylated precursors. As before, the structure of the **20 c** was confirmed to be all‐*anti* with respect to the carbazole nitrogen atoms by NMR spectroscopy (the observed high symmetry only allows for all‐*syn* or all‐*anti*), DFT calculations, and X‐ray diffraction. With 73 kcal mol^−1^ the strain energy was the same as that of [8]CPP.^[72]^ The shifts in wavelength were only minor in both absorption and fluorescence emission, although several solvents of different polarity (cyclohexane, chloroform, and THF) were used. Finally, **20 c** was used as active material in a p‐channel organic field‐effect transistor and provided a hole mobility of *μ*=1.1×10^−5^ cm^2^ V^−1^ s^−1^ with a threshold voltage of *V*_Th_=24 V and an on/off ratio of the drain current of 4.26×10^4^. This is one of few examples of nanohoops being used in optoelectronic devices.^[16]^

The Stępień group synthesized the lemniscular‐shaped [16]CPP derivative **22** with a central bicarbazole in 2019.^[65]^ Starting from a fourfold borylated bicarbazole, Jasti corners (Scheme 1 a) were attached on each functionality, and a Ni‐mediated Yamamoto macrocyclization was employed. The structure was confirmed by mass spectrometry and NMR spectroscopy, supported by DFT calculations. Although no single crystal of **22** for X‐ray diffraction could be obtained, its not fully aromatized precursor was investigated in the solid state and showed the expected connectivity. The phenylene rings in **22** had different bend angles: 12.2° for the outermost rings, comparable to [6]CPP, and 3.3° for the innermost rings next to the bicarbazole. The absorption was redshifted by 18 nm compared to [16]CPP, and the emission was even more strongly affected (496 nm vs. 415 and 438 nm in [16]CPP, Figure 6 c) with values similar to [9]CPP, yet a lower FQY of *Φ*_F_=0.36. **22** is a chiral molecule, and its enantiomers (atropisomers) were separated by chiral HPLC. No racemization at room temperature was observed due to a calculated high racemization barrier of 51.4 kcal mol^−1^. The absolute structure of the enantiomers was verified by comparison of the experimental and calculated CD spectra (Figure 6 c). A recent computational study by Si and Yang showed that functionalizing **22** with both a donor and acceptor unit reduces its HOMO–LUMO gap, modulates the HOMO/LUMO distributions, and modifies the electronic transition properties and further revealed such derivatives to be excellent candidates for second‐order nonlinear optical materials.^[73]^

## 5 Nanohoops with Other Heteroaromatics

### 5.1 N‐Containing Six‐Ring Heterocycles

**Figure 8:**
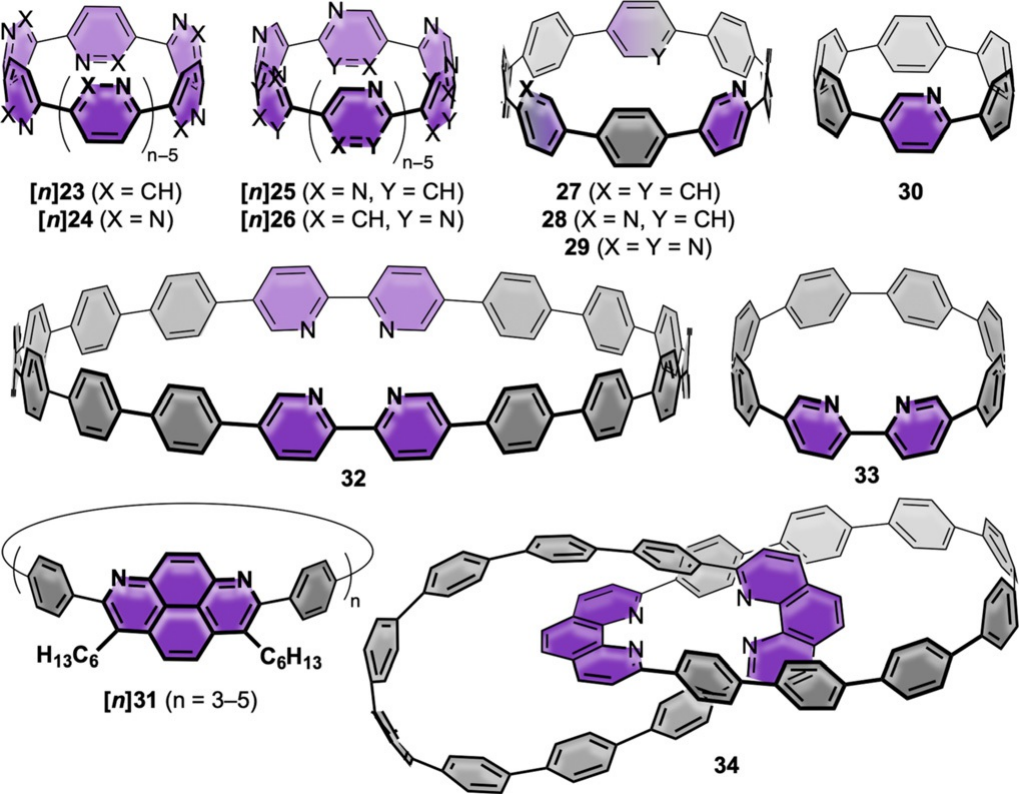
Nanohoops with N‐containing six‐ring heterocycles **[*n*]23**–**[*n*]26**,^[71]^ **27**–**29**,^[46]^ **30**,^[47]^ **32**,^[76]^ **33**,^[77]^ **[*n*]31**,^[78]^ and **34**.^[79]^

As discussed in Section 2, exchanging a benzene for a pyridine ring in [*n*]CPPs does not significantly alter their electronic properties, in spite of the more electron‐poor character of pyridine. Yet such hoops are of interest for other reasons, for instance bipyridines are excellent ligands for metal ions, which has been explored by several groups (see below). Furthermore, quaternization of the N atoms provides a handle for further functionalization. Reported nanohoops incorporating N‐containing six‐ring heterocycles are shown in Figure 8, and their optoelectronic properties are listed in Table 4. In addition, in 2020 a CPP‐based, two bipyridine‐containing lemniscal bis‐macrocycle was reported,^[74]^ and in the same year the Jasti group incorporated a *meta*‐linked pyridyl unit into a [5]CPP and used the resulting macrocycle in a metal‐templated approach to access a daisy‐chain rotaxane.^[75]^

**Table 4:** Optoelectronic properties of *N*‐heterocycle‐containing nanohoops discussed in Section 5.1.

Cmpd.	*λ*_max_ [nm]^[a]^	*ϵ* [m ^−1^ cm^−1^]	*λ*_em_ [nm]^[a]^	*Φ*_F_	*E*_1/2,Ox_ [V]^[b]^	*E*_1/2,Red_ [V]^[b]^
**32** ^[76]^	344	7.3×10^4^	427	0.80	–	–
**27** ^[46]^	345	2.8×10^4^	541	0.11	–	−2.39^[c,e]^
**28** ^[46]^	349	9.2×10^4^	544	0.13	–	−2.32^[c,e]^
**29** ^[46]^	353	1.1×10^4^	542	–	–	−2.39^[c,e]^
**30** ^[47]^	342	5.5×10^4^	–	–	0.67^[d,f]^	−2.18^[f]^
**33** ^[77]^	345	1.2×10^5^	–	–	–	–
**34** ^[65]^	327	5.4×10^5^	466	0.43	1.73^[d,f]^	−0.78,^[c or d]^ −2.58^[d]^

[a] In CH_2_Cl_2_. [b] From cyclic voltammetry vs. Fc/Fc^+^. [c] Quasi‐reversible. [d] Irreversible. [e] Cathodic peak potential. [f] Anodic peak potential.

In 2010 Bachrach and Stück performed DFT calculations on a variety of N‐containing [*n*]CPPs with incorporated pyridine (**[*n*]23**), pyridazine (**[*n*]24**), pyrazine (**[*n*]25**), or pyrimidine (**[*n*]26**) units (*n*=3–16, 18, 20, 22, 24).^[71]^ The motivation behind this work was to eliminate the *ortho*,*ortho*′ steric interaction between two neighboring phenylene rings in [*n*]CPPs by replacing CH by N. Indeed, the strain energies of most derivatives **[*n*]23**–**[*n*]26** were lower than those of the corresponding [*n*]CPPs. Depending on the substitution pattern, however, large deviations were observed. **[*n*]25** and **[*n*]26** had the lowest strain energies, up to 27 kcal mol^−1^ lower for **[5]25** than for [5]CPP. **[*n*]24** had the highest strain energies of all four compounds with a huge variation for even‐ and odd‐numbered *n*, even reaching strain energies higher than those of [*n*]CPP for *n*=13 and 15. This was due to forced unfavorable s‐*cis* conformations of the N atoms, leading to a repulsion between the nitrogen lone pairs.

As described earlier, the Jasti group synthesized the aza[*n*]CPPs **27**–**30** (*n*=6, 8).[^46^, ^47^] Only a slight redshift in the absorption was observable with the increasing number of nitrogen atoms from 340 nm ([*n*]CPPs) to 353 nm (**29**). The fluorescence maxima were also redshifted to 544 nm for **28** (533 nm for [8]CPP) with no emission for **30**, similar to [6]CPP. In accordance with the trend in [*n*]CPPs, the reduction potential of **30** was shifted by 0.21 V to higher potential compared to the aza[8]CPP **27**.

In 2016, the groups of Chiba and Isobe incorporated 1,8‐diazapyrene units into [*n*]CPPs to obtain nanohoops **[*n*]31** (*n*=3–5).^[78]^ While preliminary studies using a 2,7‐diphenylpyrene unit in a Pt‐mediated coupling reaction (Scheme 1 c) of boronic esters did not furnish any macrocycles, the introduction of nitrogen atoms into the structure allowed for the synthesis of **[*n*]31**. The nanohoops showed simple NMR spectra due to the fast conformational fluctuation of their structures, even at −60 °C. An X‐ray structure for **[4]31** provided a diameter of 21.3 Å, which is similar to that of [16]CPP with 22.1 Å.^[80]^ The azapyrene units alternately pointed upward and downward. The dihedral angle of the biphenyl units was quite large with 40–50° compared to [16]CPP (34.6°)^[71]^ or other nanohoops such as [4]cyclo‐2,8‐chrysenylene (18.5°).^[81]^

The Itami group used their cyclohexane corner unit (Scheme 1 b) and Suzuki–Miyaura coupling reactions in 2012 to obtain the 2,2’‐bipyridine‐containing [18]CPP derivative **32**.^[76]^ With a diameter of ca. 25 Å it has the same size as [18]CPP. The absorption and fluorescence maxima were slightly redshifted in comparison, as also observed for other nitrogen‐substituted CPPs (see below for aza[8]CPPs). The absolute FQY was high with *Φ*_F_=0.80. Upon protonation of the bipyridine moiety a redshift of absorption and fluorescence was observed. This is in line with Jasti's observation that methylation of the N atom creates a donor–acceptor character of the nanohoop, as discussed above for **3**–**5**. For protonated **32** the peak shape with a shoulder was similar to those of the methylated aza[8]CPPs **3** and **4**.^[46]^

**Figure 9:**
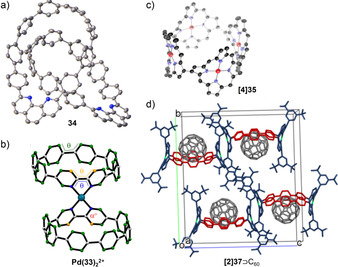
Molecular structures or packing in the solid state (thermal ellipsoids shown at 30 % probability levels, H atoms and solvent molecules omitted for clarity) of a) catenane **34**, reprinted from ref. [79], licensed under CC BY 4.0, https://creativecommons.org/licenses/by/4.0/.; b) Pd^2+^ complex of **33**, reprinted with permission from ref. [77]; copyright 2017 American Chemical Society; c) **[4]35** (substituents not shown), reprinted with permission from ref. [82]; copyright 2015 American Chemical Society, and d) C_60_ complex of **[2]37**, reprinted with permission from ref. [83]; copyright 2019 American Chemical Society.

After Itami and co‐workers had already indicated a possible Pd‐complexation through bipyridine‐containing **32**, the Jasti group followed with their own work on the smaller **33** with one bipyridine unit as a [8]CPP derivative in 2017.^[77]^ **33** was synthesized using the Jasti corner in the bent precursor (Scheme 1 a) and a Ni‐mediated coupling of the 2‐chloropyridine units as the ring‐closing step on a multigram scale. Indeed, reaction of **33** with PdCl_2_ led to a PdCl_2_‐complexed species, which could be transformed into the corresponding Pd‐complexed dicationic dimer by abstracting the chloride ions with AgBF_4_. Single‐crystal X‐ray diffraction proved a *trans* geometry of the dimeric structure at the Pd center (Figure 9 b). A Ru complex was also synthesized. While the optical properties of **33** did not significantly differ from [8]CPP (main absorption at 345 nm compared to 340 nm), the main absorptions of the Ru complex were redshifted with a broad band from 425–575 nm for the metal‐to‐ligand charge transfer.

Lastly, Zhu, Cong, and co‐workers synthesized catenane **34** in 2018, where each nanohoop of the catenane consists of eight *para*‐phenylene and one phenanthroline unit.^[79]^ Two monomeric precursors with incorporated Jasti corners (Scheme 1 a) were transformed into a catenane using Sauvage's copper(I)‐templated method.^[84]^ Ring closure was achieved using a Pd‐catalyzed oxidative homocoupling of boronic esters, followed by removal of the copper ion and aromatization. For comparison the single hoop was also synthesized. Single‐crystal X‐ray diffraction unambiguously confirmed the formation of the catenane and the Möbius topology of **34** (Figure 9 a). With a diameter of 1.1–1.4 nm, its size is comparable to [8]–[10]CPP. The highly symmetric ^1^H NMR spectrum suggested fast conformational changes. The absorption and fluorescence maxima of **34** and its single hoop were similar and showed the same fluorescence wavelength as [10]CPP of 466 nm. ACID (anisotropy of the current induced density) calculations indicated a local aromaticity in each benzene ring and the phenanthroline unit. Calculations showed that nonbonding interactions contributed −84 kcal mol^−1^ of stabilization to the catenane structure.

### 5.2 Porphyrins

**Figure 10:**
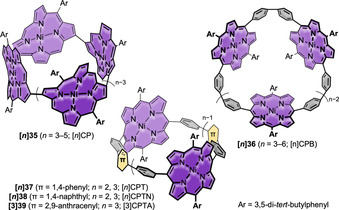
Porphyrin‐containing nanohoops **[*n*]35**,^[82]^ **[*n*]36**,^[86]^ and **[*n*]37**–**[3]39**.^[83]^

Cyclic porphyrin arrays have been of interest as models for artificial light‐harvesting antenna complexes, hosts for molecular recognition, and scaffolds for efficient hole delocalization.[^85^, ^86^] Figure 10 provides an overview of reported nanohoops containing porphyrin units, and their optoelectronic properties are summarized in Table 5. The first examples of porphyrin nanohoops solely consisting of *para*‐connected aromatic units and porphyrins were reported in the past six years. In 2015, Kim, Osuka, and co‐workers employed a Pt‐mediated coupling reaction (Scheme 1 c) to connect 1,12‐diborylated Ni‐porphyrins to form cycloporphyrins **[*n*]35** ([*n*]CP, *n*=3–5).^[82]^ The structures were highly symmetric as observed by NMR spectroscopy, and the protons showed an upfield shift with smaller hoop sizes. X‐ray diffraction of all three compounds revealed saddle conformations of the porphyrin units due to the hoop strain. The diameters were 9.32, 11.95, and 15.60 Å for **[3]35**, **[4]35**, and **[5]35**, respectively. These sizes were comparable to those of [7]CPP, [9]CPP, and [12]CPP.^[80]^ The calculated ring strain energies were up to 8 kcal mol^−1^ lower than for the parent [*n*]CPPs. The main absorption (Soret band) of the porphyrin units was redshifted with increasing size. Electrochemical investigations revealed several oxidation and reduction events. The half‐wave oxidation and reduction potentials both increased in absolute value with hoop size.

**Table 5:** Optoelectronic properties of porphyrin‐containing nanohoops discussed in Section 5.2.

Compound	*λ*_max_ [nm]^[a]^	*ϵ* [m ^−1^ cm^−1^]	*E*_1/2,Ox_ [V]^[b]^	*E*_1/2,Red_ [V]^[b]^	*E*_HOMO_ [eV]^[c]^	*E*_LUMO_ [eV]^[c]^	*E*_gap_ [eV]^[c]^
**[3]35** ^[82]^	409	2.4×105	0.36,^[d or e]^ 0.6^[d or e]^	−1.71,^[d or e]^ −1.91^[d or e]^	−5.0	−2.4	2.6
**[4]35** ^[82]^	414	3.0×10^5^	0.41,^[d or e]^ 0.58,^[e or f]^ 0.64^[e or f]^	−1.64,^[d or e]^ −1.86,^[d or e]^ −2.05^[d or e]^	−5.0	−2.5	2.5
**[5]35** ^[82]^	423	4.4×10^5^	0.46,^[d or e]^ 0.64^[d or e]^	−1.58,^[e or f]^ −1.65,^[e or f]^ −1.80^[e or f]^	−5.0	−2.4	2.6
**[3]36** ^[86]^	435	2.8×10^5^	0.37,^[e]^ 0.77^[e]^	−1.61^[e]^	−5.0	−2.3	2.7
**[4]36** ^[86]^	430	4.3×10^5^	0.47^[e]^	−1.74^[f]^	−5.1	−2.3	2.8
**[5]36** ^[86]^	428	7.5×10^5^	0.50^[d or e]^	−1.82^[f]^	−5.1	−2.2	2.9
**[6]36** ^[86]^	427	1.2×10^6^	0.51^[e]^	−1.78^[f]^	−5.1	−2.2	2.9
**[2]37** ^[83]^	446	3.4×10^5^	0.32,^[e]^ 0.68^[f]^	−1.57,^[e]^ −1.86^[f]^	–	–	–
**[3]37** ^[83]^	432	4.0×10^5^	0.45,^[d] or [e]^ 0.84^[f]^	−1.77,^[e]^ −1.98^[f]^	–	–	–
**[2]38** ^[83]^	442	2.7×10^5^	0.34, 0.60	−1.69, −1.92	–	–	–
**[3]38** ^[83]^	430	4.0×10^5^	0.44, 0.73	−1.79, −2.04	–	–	–
**[3]39** ^[83]^	428	6.2×10^5^	0.49, 0.87	−1.83, −2.10	–	–	–

[a] In CH_2_Cl_2_. [b] From cyclic voltammetry vs. Fc/Fc^+^. [c] B3LYP/6‐31G* (or with LanL2DZ for Ni). [d] Reversible. [e] Quasi‐reversible. [f] Irreversible.

Kim, Osuka, and co‐workers continued their research on conjugated porphyrin hoops in 2015 by connecting Ni‐porphyrins through biphenyl bridges in the 5,15‐positions to yield **[*n*]36** ([*n*]CPB, *n*=3–6).^[86]^ A Pt‐mediated cyclization (Scheme 1 c) of borylated precursors was used. The hoops reached diameters ranging from 16 to 33 Å with ring strain energies from 16.0 (**[6]36**) to 49.3 kcal mol^−1^ (**[3]36**), which is comparable to [*n*]CPPs of similar sizes. Interestingly, the calculated strain energies significantly increased when a demetalated (by up to 17.9 kcal mol^−1^) or Zn‐containing porphyrin unit (21.4 kcal mol^−1^) was used, and the demetalated free bases of **[*n*]36** were unstable under ambient conditions. The main absorption bands of **[*n*]36** redshifted with decreasing hoop size because of the smaller dihedral angles between the porphyrin and biphenyl units, resulting in a higher conjugation. With increasing hoop size, the oxidation potentials shifted to higher values, while the reductions shifted cathodically, therefore increasing the electrochemical HOMO–LUMO gap.

The groups of von Delius and Meyer followed in 2019 with a π‐extension of Osuka's porphyrin hoops to **[*n*]37**, **[*n*]38**, and **[3]39**.^[83]^ The hoops were synthesized using Jasti's corner unit (Scheme 1 a) and Suzuki–Miyaura coupling reactions. Synchrotron X‐ray diffraction revealed an oval‐shaped structure for the small hoop **[2]37** with a diameter of 13.2 Å. The hoop strain for this to date smallest porphyrin nanohoop amounted to 54.0 kcal mol^−1^ (B3LYP‐D3/def2‐TZVP). Absorption spectra showed the largest redshift of the porphyrin Soret‐ (30 nm) and Q‐band (40 nm) yet for **[2]37** compared to the larger hoops shown in Figure 10. With a diameter similar to [10]CPP, both **[2]37** and **[2]38** bound C_60_ with a binding constant of 3×10^8^ m ^−1^, while C_70_ was bound more strongly by **[2]38** due to its π‐extended naphthyl groups (1×10^8^ m ^−1^ vs. 2×10^7^ m ^−1^ for **[2]37**⊃C_70_). This indicates stronger binding than in [10]CPP⊃C_60_ (3×10^6^ m ^−1^ in the same solvent toluene). X‐ray diffraction showed the fullerene in **[2]37**⊃C_60_ to sit slightly above the nanohoop rim and to induce a more spherical shape in the hoop.

## 6 Nanohoops Based on Polycyclic Aromatic Hydrocarbons

In contrast to electronically modulating nanohoops, many groups set their goal on synthesizing hoops containing polycyclic aromatic hydrocarbons (PAHs) to investigate their dynamic properties, to use them as model segments for carbon nanotubes, or to induce chirality in the hoops. Several examples were published for smaller PAHs, such as naphthalene and anthracene, as well as larger structures, such as hexabenzocoronene. In many cases VT‐NMR measurements or CD spectroscopy was used to investigate rotational barriers of the bent PAHs through the hoop. In this section we will discuss PAH‐containing nanohoops sorted by the type of the PAH incorporated. The optoelectronic properties of these nanohoops can be found in Table S1 in the Supporting Information.

### 6.1 Nanohoops Incorporating Five‐Ring‐Containing PAHs

**Figure 11:**
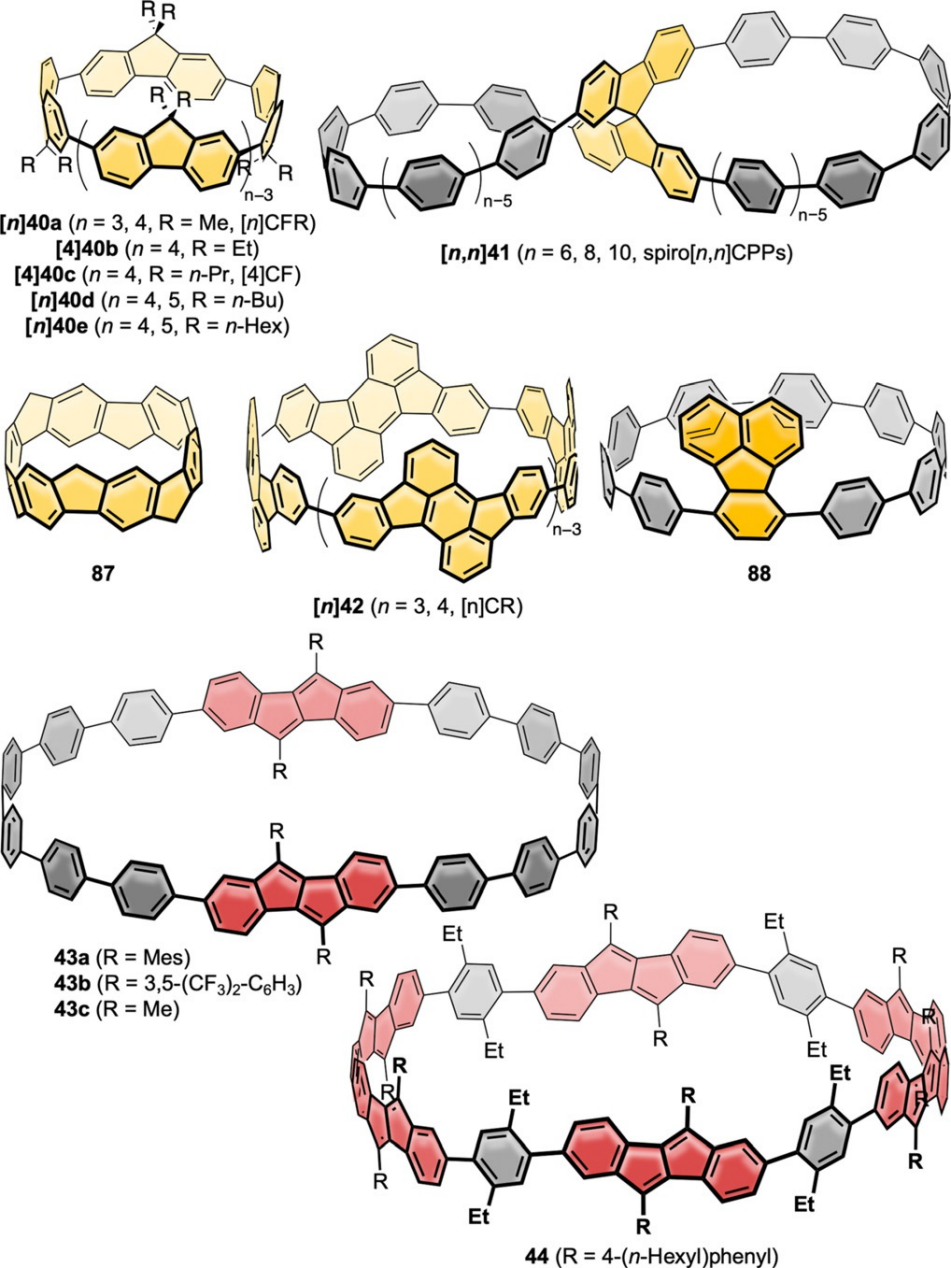
Nanohoops with five‐ring‐containing PAHs **[*n*]40 a**,^[87]^ **[4]40 b**,^[89]^ **[4]40 c**,^[88]^ **[*n*]40 d** and **e**,^[90]^ **[*n***, ***n*** **]41**,^[74]^ **87**,^[91]^ **[*n*]42**,^[92]^ **88**,^[93]^ **43**,^[94]^ and **44**.^[40]^ The color red is used to highlight the dibenzo[*a*,*e*]pentalene moieties due to their small band gap and red color.

Fluorene is one of the most abundant co‐monomers in conjugated co‐polymers for OLED applications. The bridging of two phenyl rings by a saturated carbon increases rigidity and conjugation, leading to a redshift in absorption and emission and a small Stokes shift compared to biphenyl. There are several reports of nanohoops with incorporated fluorene groups (Figure 11). In the first example from 2015, Yamago and co‐workers synthesized **[3]‐** and **[4]40 a**.^[87]^ In contrast to most nanohoops synthesized via the Pt‐mediated route (Scheme 1 c) they obtained a trinuclear in addition to the tetranuclear Pt intermediate by changing the solvent and Pt precursor in the cyclization step. This was possible due to the geometry of the fluorene moiety. **[3]40 a** was received as a mixture of two rotamers, which were separable by chiral HPLC, while **[4]40 a** was only observed as the all*‐anti* rotamer shown in Figure 11. This was due to a high calculated rotational barrier of the fluorene units in **[3]40 a** of 58 kcal mol^−1^, not allowing interconversion even at 180 °C, as shown by VT‐NMR. In comparison, **[4]40 a** has a low barrier of 18 kcal mol^−1^, theoretically allowing for dynamic conformational changes. The higher rigidity of **[*n*]40 a** compared to [*n*]CPPs led to an enhanced conjugation and bathochromic shift of the absorption maxima as well as a cathodic shift of the first oxidation potentials compared to [6]‐ and [8]CPP. Most noteworthy is the significantly enhanced FQY of *Φ*_F_=0.32 for **[4]40 a** compared to [8]CPP (*Φ*_F_=0.081) together with a smaller Stokes shift due to this rigidity. Similar to these hoops is **[4]40 c** with propyl side chains, synthesized by Loh, Huang, and co‐workers and published in 2016.^[88]^ Here, also Yamago's procedure using a Pt‐mediated route (Scheme 1 c) was used. NMR spectroscopy and single‐crystal X‐ray diffraction revealed the all*‐anti* rotamer with a slightly oval hoop shape (*d*: 1.19×1.00 nm). Both in solution and in thin film **[4]40 c** showed an emission at 512 nm with an even higher FQY than **[4]40 a** of *Φ*_F_=0.45. To prove its practical applicability, they used **[4]40 c** as the emitter in an OLED showing strong green emission with a brightness of 878 cd cm^−2^ at 10 V and a maximum luminescence efficiency of 0.83 cd A^−1^. This was one of the first examples of a nanohoop being used in an optoelectronic device.^[16]^

In 2018 Quinton, Poriel, and co‐workers reported **[4]40 b** with ethyl side groups on the fluorene units, synthesized in a Pt‐mediated route (Scheme 1 c).^[89]^ X‐ray crystallography revealed the hoop to possess an all‐*anti* conformation, as had been reported for the other [4]cyclofluorenes discussed above. Interestingly, compared to the emission maxima of **[4]40 a** (514 nm in CHCl_3_) and **[4]40 c** (512 nm in THF), a blueshift by 22 nm was observed, resulting in an emission maximum of 492 nm (in CHCl_3_) for **[4]40 b**. This was most likely due to the structural arrangement of the fluorene units because of the change in alkyl chain length. In 2020 the Poriel group extended the cyclofluorene family to *n*=5 with butyl‐ and hexyl‐substituted derivatives **[*n*]40 d** and **[*n*]40 e** (with *n*=4, 5).^[90]^ A Pt‐mediated synthetic route was chosen, which likely furnished the five‐membered product in addition to *n*=4 due to the longer alkyl side chains on the fluorene units. The molecular structures of **[4]‐** and **[5]40 d** were resolved by X‐ray diffraction.

In 2020 Schaub et al. incorporated spirobifluorene units into CPPs, resulting in the figure‐eight‐shaped, lemniscal bismacrocycles **[*n***, ***n*** **]41**, so‐called spiro[*n*,*n*]CPPs (*n*=6, 8, 10).^[74]^ The syntheses started from a tetrabromospirobifluorene, which was coupled in a Suzuki–Miyaura reaction with two C‐shaped units of different sizes containing two Jasti corners each (Scheme 1 a). In addition, a derivative of **[6,6]41** was synthesized containing four pyridine units in the outer loops. Three of the lemniscal bis‐macrocycles were characterized by single‐crystal X‐ray diffraction. Compared to [*n*]CPPs (*λ*_max_=335–340 nm), the absorption maxima were redshifted to *λ*_max_ 353–358 nm. The fluorescence blueshifted with increasing size (from 493 nm for *n*=8 to 454 nm for *n*=12), which is a common feature for CPP‐derived macrocycles. Due to their porous solid‐state structures, the gas and vapor analyte uptake of **[*n***, ***n*** **]41** was investigated and showed an increased guest uptake compared to [*n*]CPPs.

In 2020 Itami and co‐workers reported [6]CPP derivative **87**, a compound related to fluorene‐containing nanohoops, where each phenylene unit is linked to its neighbors through methylene bridges, resulting in a belt‐shaped structure.^[91]^ A pillar[6]arene was used as the synthetic precursor, which already possessed the required hexagonal geometry. The additional aryl—aryl linkages were introduced via Ni‐mediated coupling of triflates. The structure of **87** was confirmed by X‐ray crystallography. Due to its more rigid structure, the HOMO energy of **87** was higher than that of [6]CPP (oxidation potential 0.03 V compared to 0.44 V, respectively, both vs. Fc/Fc^+^) and the band gap was smaller.

**Figure 12:**
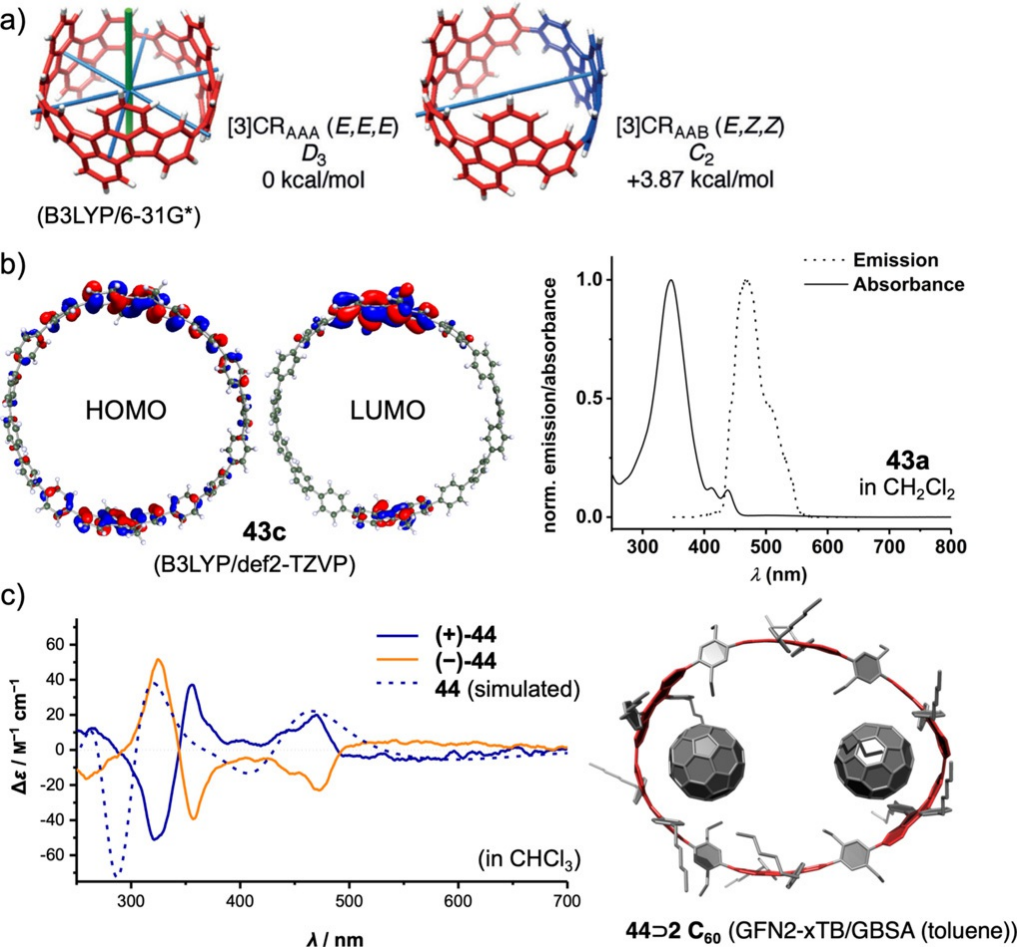
a) Two possible diastereomers of **[3]42**, reprinted with permission from ref. [92]; copyright 2017 Wiley‐VCH; b) frontier molecular orbitals of **43 c** (with R=Me) and optical spectra of **43 a**, reprinted with permission from ref. [94]; copyright 2020 American Chemical Society; c) electronic circular dichroism spectra of **(+)‐44** and **(−)‐44** with spectrum simulated for enantiomer **(+)‐44** and calculated equilibrium structure of **44**⊃2 C_60_, reprinted with permission from ref. [40], copyright 2020 Wiley‐VCH GmbH.

In contrast to fluorene with a partially saturated five‐membered ring, PAHs with conjugated five‐membered rings can significantly alter the electronic properties of the hoops due to their non‐alternant π‐system. Four examples exist for such PAHs incorporated into nanohoops, namely rubicene (**[*n*]42**), fluoranthene (**88**), and dibenzo[*a*,*e*]pentalene (DBP, **43** and **44**) (Figure 11). The cylinder‐shaped nanohoops **[*n*]42** ([*n*]CR) consisting of rubicene units were synthesized by Isobe (2017) using the Pt‐mediated cyclization (Scheme 1 c).^[92]^ Both the three‐ and four‐membered congeners were isolated (*n*=3, 4). All four possible diastereomers of **[4]42** could be separated by chiral HPLC, while **[3]42** was formed as one of two possible diastereomers (Figure 12 a) with all‐*syn* orientation of the rubicene groups, as confirmed by NMR spectroscopy and X‐ray diffraction. UV/Vis spectra indicated conjugation around the hoop and an extended π‐system compared to monomeric rubicene. This was in line with a bond length evaluation, which indicated reduced aromaticity and an enhanced quinoidal character for **[3]42** compared to rubicene.

In 2020 the Du group reported [10]CPP derivative **88** with an incorporated fluoranthene group.^[93]^ Synthesis was afforded using a C‐shaped synthon for a [7]paraphenylene linker based on three Jasti corner units (Scheme 1 a) in a Suzuki–Miyaura reaction with a bis‐borylated diphenylfluoranthene unit. While the absorption spectrum strongly resembled that of [10]CPP, the emission maximum was redshifted by 22 nm to 488 nm with an FQY of *Φ*_F_=0.49. **88** can host one C_60_ molecule with a binding constant of 1.4×10^6^ m ^−1^, which is similar to that of [10]CPP (3×10^6^ m ^−1^ in the same solvent toluene).

In 2020 our group published the first synthesis of a nanohoop containing an antiaromatic system, namely **43**, with two dibenzo[*a*,*e*]pentalene units.[^94^, ^95^] The synthesis included using the Itami corner unit (Scheme 1 b) and a Yamamoto cyclization followed by oxidative aromatization to yield two electronically different derivatives **43 a** and **43 b**. In accordance with previous results from our group, bending the DBP slightly reduced its antiaromaticity.^[96]^ The redox properties reflected the ambipolar character of the DBP units with reversible oxidations and reductions and the expected electronic impact of the substituents R. The frontier molecular orbitals were separated between the DBP units and the *para*‐phenylene linkers with electron densities in HOMO−1, HOMO, LUMO, and LUMO+1 mainly localized on the DBPs (Figure 12 b). The presence of these two π‐subsystems was also reflected in an additive way in the UV/Vis absorption spectra. **43 a** was emissive due to the *para*‐phenylene linkers, however, with a FQY of <1 % due to energy or charge transfer to the non‐emissive DBP units.

In 2021 we followed up with chiral DBP‐based nanohoops **44** accessed in a stereoselective synthesis.^[40]^ Both enantiomers **(+)‐** and **(−)‐44** were synthesized separately by using enantiomerically pure and bent diketone precursors (Scheme 1 f). The small HOMO–LUMO gap and ambipolar electrochemical character of the DBP units were reflected in the optoelectronic properties of the hoops. Electronic circular dichroism spectra measured at elevated temperatures and molecular dynamics simulations showed that **(+)‐44** did not racemize even when heated to 110 °C in spite of its conformational flexibility regarding the outer shape. Due to its large diameter of ca. 2.5 Å, **44** was able to accommodate two C_60_ molecules, as NMR‐based binding studies showed (Figure 12 c).

### 6.2 Nanohoops Incorporating Six‐Ring‐Based PAHs

#### 6.2.1 Naphthalene

**Figure 13:**
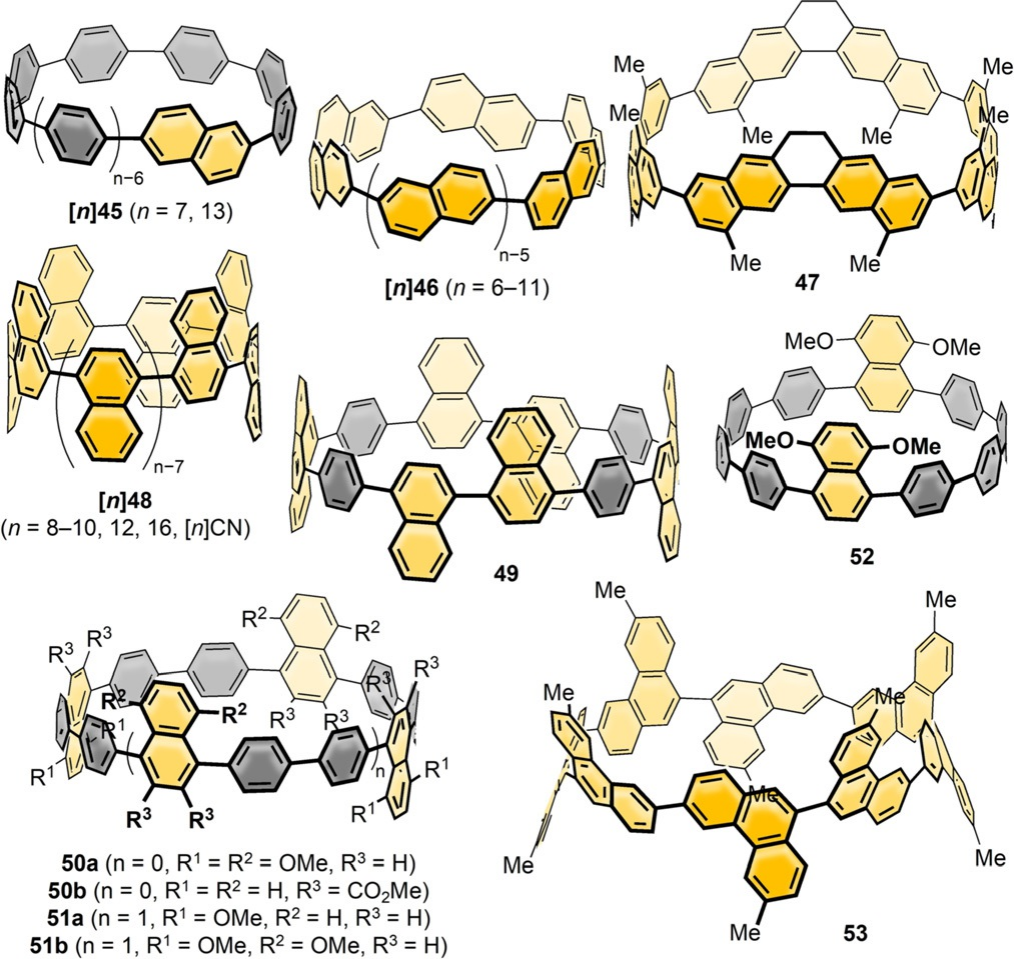
Naphthalene‐containing nanohoops **[*n*]45**,[^97^, ^98^] **[*n*]46**,[^100^, ^101^] **47**,^[100]^ **[*n*]48**,[^102^, ^103^, ^104^] **49**,^[105]^ **50 a**,^[38]^ **50 b**,^[57]^ **51** and **52** ^[106]^ and phenanthrene‐containing hoop **53**.^[107]^

Naphthalene is the PAH most often incorporated into nanohoops. The reported examples are shown in Figure 13. Shortly after their first synthesis of [*n*]CPPs, Itami and co‐workers published the synthesis of a (2,6)‐naphthalene‐containing [13]CPP, namely **[13]45**.^[97]^ They used the Itami corner unit (Scheme 1 b) and Suzuki–Miyaura couplings for ring closure. Although **45** possessed helical chirality, the calculated racemization barrier was low with 8.4 kcal mol^−1^ due to the large size of the hoop. In 2019 Du's group used a C‐shaped synthon for a [7]paraphenylene linker based on three Jasti corner units (Scheme 1 a) in a Suzuki–Miyaura reaction with a 2,6‐bifunctionalized naphthalene to synthesize the more strained **[7]45**.^[98]^ Its main absorption at 335 nm was comparable to the absorption maxima of [*n*]CPPs. The vibrational structure of the emission spectrum of **[7]45** resembled that of the larger [10]–[12]CPPs while being redshifted due to the lower optical band gap. The FQY (*Φ*_F_=0.30) lay between those of [8]‐ and [9]CPP. Another hoop consisting of alternating aryl‐ and 2,6‐connected naphthyl units (six each) was published by Chi, Miao, and co‐workers in 2019.^[99]^ It served as a precursor to a conjugated nanobelt **83** (see Figure 23 in Section 7), and no optoelectronic data were published.

In 2015 Isobe's group published the first synthesis of a 2,6‐connected, solely naphthalene‐containing hoop (**[8]46**) using Yamago's route (Scheme 1 c) with diborylated binaphthyl units.^[100]^ The naphthyl units in **[8]46** rapidly rotated at room temperature due to a low calculated barrier of 10.5 kcal mol^−1^. Three out of 18 possible diastereomers of **[8]46** were crystallized by changing the crystallization solvent and characterized by X‐ray diffraction. They also synthesized analogue **47** with bridged binaphthyl units, introducing more rigidity into the hoop and influencing its dynamic behavior. The binaphthyl units did not rotate in solution on the NMR timescale. Indeed, **47** had a redshifted absorption, indicating a higher degree of conjugation. In a later work the Isobe group synthesized five further derivatives of **[*n*]46** with *n=*6–11.^[101]^ They combined precursors of different sizes in a Yamago nanohoop synthesis (Scheme 1 c). A mathematical model was evaluated to describe the number of possible stereoisomers. With an increasing number of repeating units, the number of possible diastereomers increased up to 63 for *n=*11. For *n=*7, out of nine possible diastereomers, two (in pairs of enantiomers) were characterized in the solid state, which crystallized in two sets of disordered structures. The calculated rotational barriers ranged from 38.5 kcal mol^−1^ for *n=*4 to 5.8 kcal mol^−1^ for *n=*11.

**Figure 14:**
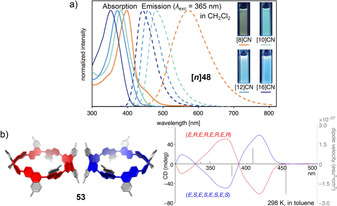
a) Optical spectra of **[*n*]48** (*n*=8, 10, 12, 16), reprinted from ref. [103], “Synthesis and properties of [8]‐, [10]‐, [12]‐, and [16]cyclo‐1,4‐naphthylenes”, licensed under CC BY 3.0, https://creativecommons.org/licenses/by/3.0/ published by The Royal Society of Chemistry. b) Molecular structure of **53** in the solid state as pair of enantiomers and CD spectra of isolated enantiomers, reprinted with permission from ref. [107], https://pubs.acs.org/doi/10.1021/acscentsci.6b00240 (further permissions related to the material excerpted should be directed to the ACS).

Examples of 1,4‐connected naphthyl groups are more abundant. In 2012 the Itami group published the synthesis of the 1,4‐connected, nine‐naphthyl‐unit‐containing hoop **[9]48** ([9]CN) by applying an adapted cyclohexadiene‐fused corner unit (Scheme 1 a) in a Ni‐mediated “shotgun” cyclization.^[102]^ They observed a dynamic conformational change that was slow on the NMR time scale, which corresponded well with the calculated rotational barrier of 21 kcal mol^−1^. The absorption maximum was redshifted (378 nm) compared to [9]CPP (339 nm), but emission occurred at almost the same wavelength with a lower FQY of *Φ*_F_=0.35. Further research on 1,4‐connected **[*n*]48** was done by the Itami (2017)^[103]^ and Du groups (2018).^[104]^ While Itami proved the applicability of his synthesis for even‐numbered **[*n*]48** with *n*=8, 10, 12, and 16, Du showed that it was also possible to use Yamago's Pt‐mediated route (Scheme 1 c) for *n*=8, 9, and 12 with a slightly higher yield in the last strain‐inducing step. There are several trends observable from decreasing *n*. The optical band gap decreased for smaller *n* with increasing strain, and the emission maxima were shifted to higher wavelengths, which is in agreement with theoretical predictions and the observations made for [*n*]CPPs (Figure 14 a). A kinetic study (via VT‐NMR) on the thermal conversion of the *C*_S_‐symmetric **[10]48** into the more stable *D*_5*d*_ conformer revealed an isomerization barrier of 27.1 kcal mol^−1^, consistent with the theoretically predicted isomerization barrier. Du's results supported these findings with only minor variations of the spectroscopic values.

In 2013 Swager and Batson reported the synthesis of hoop **49** as a [12]CPP analogue by applying Itami's corner units (Scheme 1 b) and a shotgun cyclization approach.^[105]^ Spectroscopic characterization of the hoop revealed a high conjugation throughout the system. The aim of their research was to apply the hoop as a template for CNT growth.

In 2014 Wang and co‐workers introduced their Diels–Alder synthesis of cyclohexa‐1,4‐dienes (Scheme 1 d), enabling the synthesis of many different nanohoops.^[38]^ In this publication they presented a dihydronaphthalene corner unit and a subsequent Ni‐mediated “shotgun” cyclization leading to hexamethoxy‐substituted **50 a** as a [9]CPP analogue. NMR spectra revealed a rapid rotation of the naphthalene moieties at room temperature. The absorption and fluorescence maxima of **50 a** were both redshifted by 18 nm to 362 nm and 512 nm, respectively, in comparison to parent [9]CPP. The Wang group also introduced these dimethoxynaphthalene units into hoops together with thiophenes^[60]^ and furans,^[61]^ as was discussed in Section 4.1 (Figure 5).

Nanohoop **50 b**, another [9]CPP analogue with electron‐withdrawing substituents, was published by the Wang group in 2016 and was synthesized following a similar strategy.^[57]^ X‐ray diffraction showed that the ester groups in **50 b** all canted towards the inner plane of the [9]CPP and the naphthyl groups were oriented in a *syn*,*anti* fashion. With 346 nm the absorption maximum of **50 b** was similar to [9]CPP (340 nm), while the fluorescence maximum at 445 nm was blueshifted by 49 nm compared to the latter. In 2017 the Wang group extended their synthetic route to naphthalene‐containing hoops **51** and **52**.^[106]^ **51 b** is the tetrameric congener of **50 a**. An NMR analysis showed a time‐averaged *D*_4*h*_‐symmetric structure for **51 b**, while DFT calculations predicted the all‐*anti* conformer to be the most stable. Both **51 a** and **b** had an absorption maximum at 358 nm, redshifted by 20 nm compared to parent [12]CPP, while for smaller **52** the absorption maximum at 336 nm was similar to that of parent [10]CPP. A similar redshift of 34 nm compared to [12]CPP was observed in the fluorescence of **51 a** and **b** at 484 nm, while **52** experienced a blueshift to 453 nm (vs. 470 nm for [10]CPP).

#### 6.2.2 Phenanthrene

Further research into the stereoisomerism of hoop‐shaped molecules was conducted by Isobe's group, who investigated the dynamic behavior of the phenanthrene‐based nanohoop **53** in 2016 (Figure 13).^[107]^ It can be regarded as a derivative of the previously mentioned cyclonaphthylenes with annelated benzene rings in the periphery of the hoop. **53** was synthesized using Yamago's route (Scheme 1 c) with biphenanthrene monomers. X‐ray diffraction showed a racemic mixture of the (*E*,*R*,*E*,*R*,*E*,*R*,*E*,*R*) (shown in Figure 13) and (*E*,*S*,*E*,*S*,*E*,*S*,*E*,*S*) isomers (Figure 14 b) with a time‐averaged *D*_4_ symmetry, as shown by NMR spectroscopy. In VT‐NMR measurements no splitting of the resonances was observed (see also **[*n*]46** above). The two *D*_4_‐symmetric enantiomers were separated by chiral HPLC. A VT‐CD spectroscopic measurement revealed rapid racemization between 30 and 60 °C. The racemization barrier (Δ*H* ^≠^=25 kcal mol^−1^) was in the range of that observed for 1,1′‐binaphthyl (Δ*H* ^≠^=22 kcal mol^−1^).

#### 6.2.3 Chrysene

**Figure 15:**
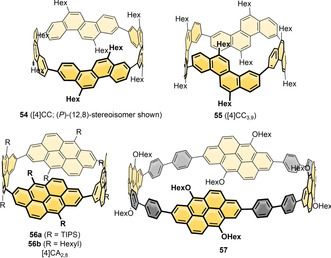
Chrysene‐ (**54** ^[108]^ and **55** ^[109]^) and anthanthrene‐containing nanohoops (**56** ^[110]^ and **57** ^[111]^).

In 2011 the Isobe group synthesized nanohoops **54** ([4]CC) with four 2,8‐connected chrysene panels using Yamago's method (Scheme 1 c, Figure 15).^[108]^ Due to their chirality they can serve as models for SWNTs. All six stereoisomers (two pairs of enantiomers and two meso‐compounds) were separated and characterized by chiral HPLC. The authors explored the possibility of asymmetric induction and were able to obtain an enantiomeric excess of 11 % and 17 % for one set of the diastereomeric pairs of enantiomers by carrying out the reductive elimination in the presence of cholesteryl stearate. In a later publication the authors investigated the fullerene complexation of one single enantiomer ((*M*)‐(12,8)) of **54** by synchrotron X‐ray diffraction, among others,^[81]^ and furthermore elucidated the solid‐state structure of the racemic mixture of the (12,8)‐isomers of **54**.

**Figure 16:**
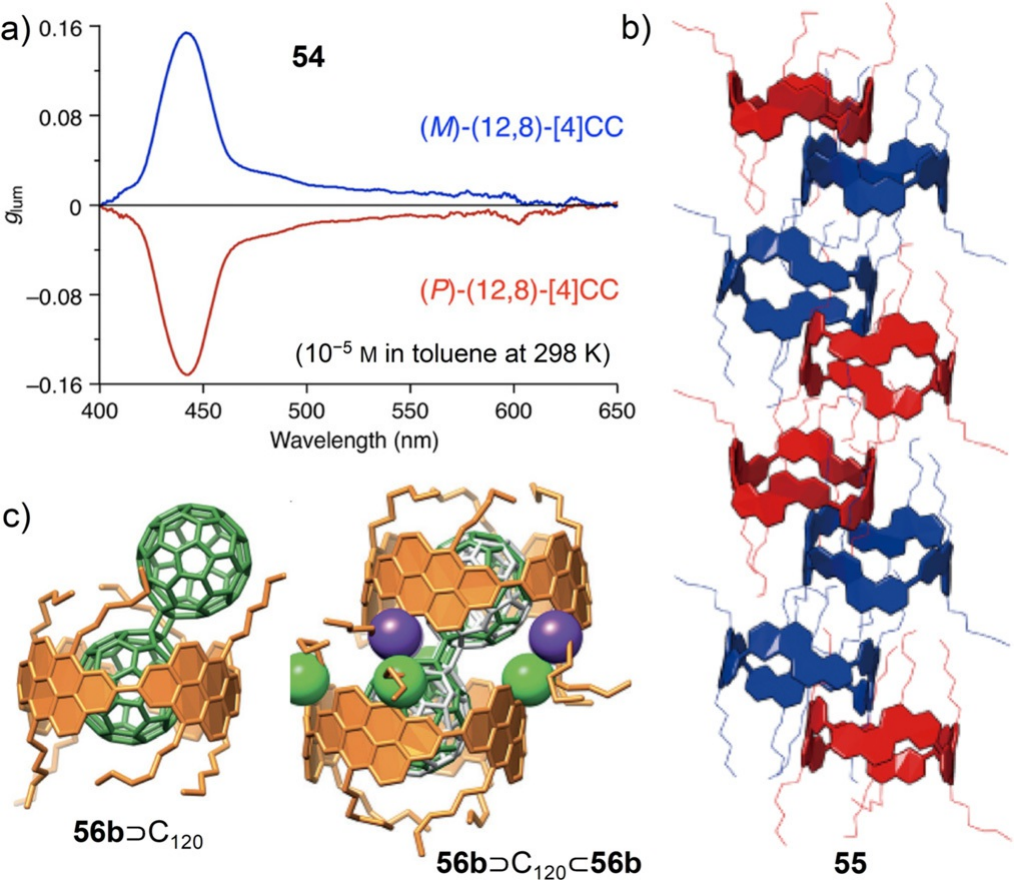
a) Circularly polarized luminescence spectra of the two enantiomers of (12,8)‐**54**, reprinted with permission from ref. [112]. b) Molecular packing of (16,0)‐**55** in the solid state in pairs of enantiomers, reprinted with permission from ref. [109]; copyright 2012 American Chemical Society. c) Molecular structures in the solid state of the C_120_ complexes of **56 b**, reprinted with permission from ref. [113]; copyright 2017 Wiley‐VCH.

In 2017 the Isobe group investigated the separate stereoisomers of **54** in more detail; among others they structurally characterized the (*P*)‐ and (*M*)‐(12,8) isomer and performed CD spectroscopic as well as circularly polarized luminescence measurements (Figure 16 a).^[112]^ A high luminescence dissymmetry factor of *g*_lum_=−0.152 was measured in conjunction with a high FQY of *Φ*_F_=0.80, which suggests that organic molecules could rival lanthanide complexes in terms of circularly polarized luminescence performance.

In 2017 the complexation of the fullerene dimer C_120_ by the (12,8) isomers of **54** as well as cycloanthanthrenylenes **56** (see Section 6.2.4) was investigated by Isobe's group.^[113]^ Two nanohoops can bind to one C_120_, resulting in a dumbbell‐shaped molecule, with association constants of *K*_a1_=1.4×10^9^ m ^−1^ and *K*_a2_=4.5×10^5^ m ^−1^ for the first and second binding event (in 1,2‐dichlorobenzene). A crystal structure of (12,8)‐**54**⊃C_120_⊃(12,8)‐**54** was also obtained.

In 2012 the Isobe group reported regioisomers **55** ([4]CC_3,9_), in which four chrysenes are connected via their 3,9‐positions.^[109]^ The synthesis proceeded using Yamago's method (Scheme 1 c) and boronic ester precursors. One out of four possible diastereomers was isolated, the *D*_4_‐symmetric (16,0) derivative, and structurally characterized by X‐ray diffraction (Figure 16 b). Chiral HPLC was used to separate the two enantiomers, which did not racemize up to 200 °C.

#### 6.2.4 Anthanthrene

The Isobe group incorporated anthanthrene panels into two different nanohoop architectures (Figure 15). The first example from 2013 was **56**, a cyclic 2,8‐connected tetramer, which was synthesized from borylated precursors using Yamago's method (Scheme 1 c).^[110]^ **56 a** with TIPS substituents was formed as the *D*_4_‐symmetric isomer shown in Figure 15 out of four possible diastereomers, as shown by X‐ray diffraction. For **56 b** with hexyl substituents, all four diastereomers formed and were separated by chiral HPLC. The rotational barrier for the anthanthrene units in **56 b** was experimentally determined to be 21 kcal mol^−1^. An association study from 2017 using the (12,8) diastereomer of **56 b** with the fullerene dimer C_120_ provided association constants of *K*_a1_=6.9×10^8^ m ^−1^ and *K*_a2_=3.2×10^3^ m ^−1^ for the first and second binding event (in 1,2‐dichlorobenzene).^[113]^ An X‐ray crystallographic structure of both the single and the double complex was obtained (Figure 16 c). In 2015 Isobe's group synthesized the larger nanohoop **57** with incorporated biphenyls using Yamago's method (Scheme 1 c).^[111]^ Due to its larger size, the structure of **57** rapidly fluctuated among the four possible diastereomers on the NMR timescale. DFT calculations provided a small rotational barrier of 8 kcal mol^−1^.

#### 6.2.5 Anthracene

**Figure 17:**
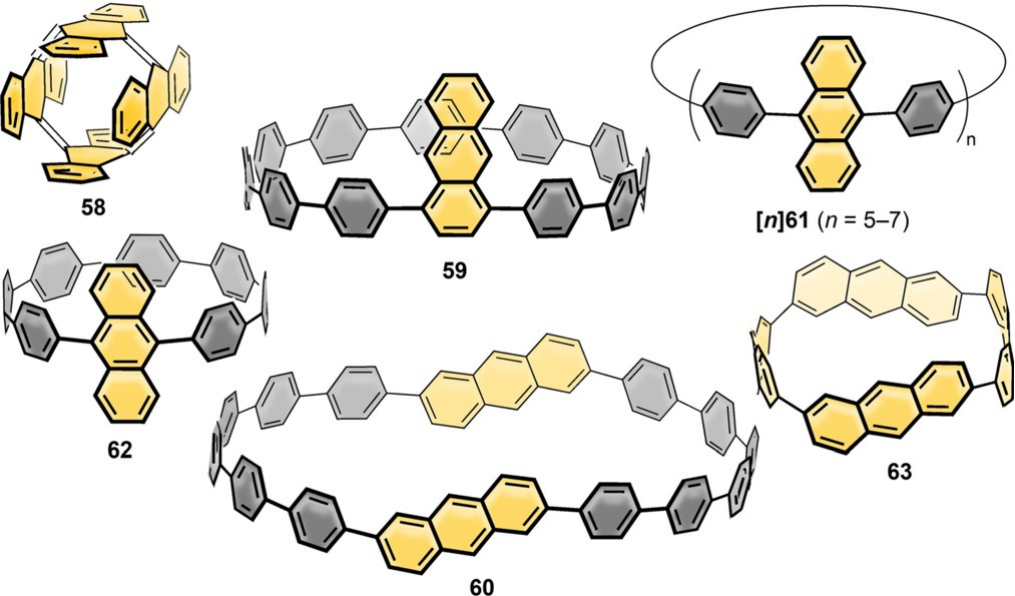
Anthracene‐containing nanohoops **58**,^[114]^ **59**,^[115]^ **60**,^[116]^ **[*n*]61**,^[39]^ **62**,^[117]^ and **63**.^[118]^

Due to its optical applications and Diels–Alder‐reactivity, several groups introduced anthracene into carbon nanohoops. In 1996, long before the first synthesis of [*n*]CPPs, the Herges group reported “picotube” **58**, which is a cyclic anthracene tetramer and [4]CPP derivative (Figure 17).^[114]^ It was synthesized by a photochemically induced ring‐expanding metathesis reaction from tetradehydrodianthracene. NMR spectroscopy and X‐ray diffraction confirmed the high *D*_4*h*_ symmetry with a diameter of 5.4 Å and a length of 8.2 Å. In spite of its strained structure, **58** was extraordinarily stable and unreactive.

Jasti's approach in 2016 was to introduce anthracene into a [12]CPP.^[115]^ 9,10‐Dihydroanthracene was coupled with a Jasti corner unit (Scheme 1 a). Subsequent ring closure by Suzuki–Miyaura reaction followed by aromatization of the ring as well as the anthracene unit gave nanohoop **59**. Its structure was confirmed by X‐ray diffraction. The authors compared **59** to a nonconjugated cyclic as well as to an acyclic anthracene derivative. The optical band gap increased in this order from 2.72 to 2.78 and 2.98 eV with a lower degree of electron delocalization, and **59** showed a lower oxidation potential of 0.65 V vs. Fc/Fc^+^ compared to the reference compounds and to [12]CPP. The electron density in the HOMO and LUMO was mainly localized on the diphenylanthracene unit. **59** did not undergo photodimerization of the anthracene unit, but Diels–Alder reactions with strong dienophiles were possible.

Other groups made use of anthracene's unique reactivity towards cycloadditions. In 2016 the Yang and Cong groups synthesized nanohoop **60** incorporating two anthracene moieties by applying a photodimerization–cycloreversion strategy.[^23^, ^116^] They coupled photodimerized anthracene with the Jasti corner unit (Scheme 1 a). Subsequent ring closure and aromatization led to a propeller‐shaped dual‐hoop, which, when heated, underwent a [4+4]‐cycloreversion and was transformed into **60**. X‐ray diffraction of the propeller‐shaped dual‐hoop revealed a racemic mixture of two enantiomers with the “propeller blades” being almost orthogonal to each other. NMR experiments as well as theoretical calculations showed a low rotational barrier (8.6 kcal mol^−1^) for the anthracene units in comparison to previously mentioned naphthylene nanohoops, leading to a rapid interconversion of the two possible conformers. This cycloreversion strategy of the precursor to **60** was used in a later work by Cong and co‐workers to synthesize pentiptycene‐bridged dual nanohoops.^[119]^ The central bridging aryl ring of the pentiptycene unit was introduced by two subsequent aryne [4+2]‐cycloadditions to the anthracene units.

Three further anthracene‐containing nanohoops **[*n*]61** were published in 2017 by Tokuyama, Isobe, and co‐workers.^[39]^ For this purpose, the new corner unit 9,10‐epoxyanthracene was used with a relatively large directing angle of 126° (Scheme 1 e) and a Ni‐mediated coupling for macrocyclization. The authors were able to crystallize two of the nonconjugated cyclic precursors (*n*=5, 7) as well as **[5]61**, which at the time was one of the largest nanohoops to be structurally determined by X‐ray diffraction. The dihedral angles between anthracenes and neighboring phenylene rings were found to be large, with 61°–89°. This was, according to theoretical calculations, the thermodynamic minimum. NMR spectroscopy revealed a highly symmetric structure with time‐averaged *D*_5*h*_ symmetry. The rotational barrier for the 9,10‐connected anthracene groups was 10 kcal mol^−1^, which is slightly higher than that for the 2,6‐substituted anthracene nanohoop **60** (see above). The similarly connected anthracene nanohoop **62** was published by the Gaeta and Peluso groups shortly thereafter.^[117]^ The synthesis was based on the Jasti approach (Scheme 1 a) with a Suzuki–Miyaura coupling for cyclization. The smaller hoop diameter hindered the rotation of the anthracene unit and led to a significant distortion of its central six‐membered ring, shown by calculations. **62** showed absorptions attributable to the diphenylanthracene unit as well the [8]CPP frame. The emission, however, significantly differed from that of [8]CPP with a broad maximum at 485 nm (*Φ*_F_=0.47). In the presence of a porphyrin Pd^II^ complex as sensitizer, **62** showed visible light up‐conversion.

**Figure 18:**
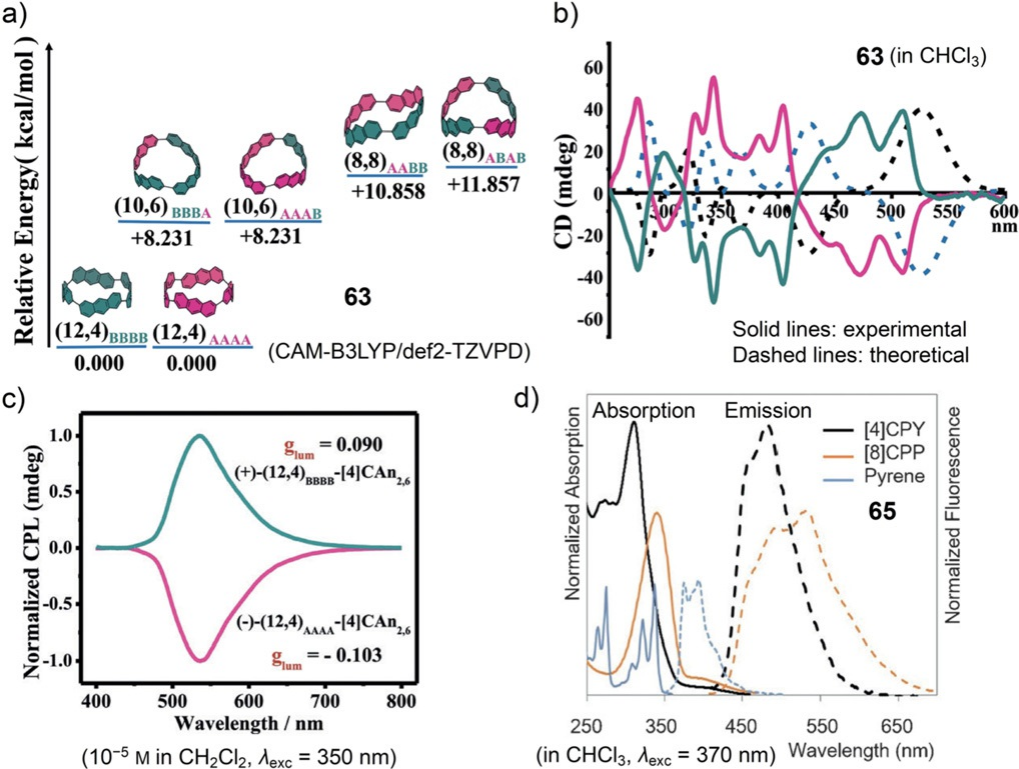
a) Calculated diastereomeric structures and energies of **63**. b) Experimental and theoretical CD and c) circularly polarized luminescence spectra of **63**, reprinted with permission from ref. [118]; copyright 2020 Wiley‐VCH. d) Optical spectra of **65**, reprinted with permission from ref. [120]; copyright 2020 Wiley‐VCH.

Apart from the Herges group's picotube **58**, the first solely anthracene‐containing nanohoop was reported in 2020 by the Du group.^[118]^ The authors proved the versatility of Yamago's Pt‐mediated approach (Scheme 1 c) and synthesized [4]cyclo‐2,6‐anthracene (**63**). The compound was obtained in a good yield of 59 % over the last two steps. The ^1^H NMR spectrum featured four signals, indicative of *D*_4_ symmtery, and no signal splitting occurred between −80 °C and 125 °C. Rotation of the anthracene units was hindered due to the small hoop size, and the two enantiomers of the most symmetric *D*_4_ conformer (shown in Figure 17) were separated by chiral HPLC. Their configurational stability was further confirmed by spectroscopic methods and theoretical calculations (Figure 18 a). In comparison to anthracene, a redshift of the longest wavelength absorption was observed owing to the larger cyclic π‐conjugated system. The pronounced emission bands were also shifted to lower energy with a FQY of *Φ*_F_=0.18. The hoop‐shaped structure of **63** was confirmed by scanning tunneling microscopy on Au(111). Circularly polarized luminescence provided sizeable dissymmetry factors *g*_lum_ of 0.103 and 0.090 for the two enantiomers (Figure 18 c).

#### 6.2.6 Pyrene

**Figure 19:**
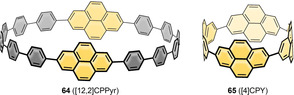
Pyrene‐containing nanohoops **64** ^[121]^ and **65**.^[120]^

Incorporating pyrene units into CPPs leads to their vertical extension as subunits of armchair carbon nanotubes. Two examples have been reported (Figure 19). The two‐pyrene‐unit‐containing hoop **64** was synthesized by the Itami group in 2014 as a [16]CPP analogue.^[121]^ Pyrene was coupled with the Itami corner unit (Scheme 1 b), cyclized in a Yamamoto fashion, and the hoop aromatized. NMR spectroscopy showed **64** to be highly symmetric. Poor conjugation between the *para*‐phenylene and the pyrenylene moieties was observed in the absorption spectra as well as in theoretical calculations. The UV/Vis absorption spectrum was a combination of the pyrene and [16]CPP bands, and DFT calculations showed orbital separation between the two. Nanohoop **64** showed a redshifted emission at 430 nm (*Φ*_F_=0.21) compared to pyrene and [16]CPP.

In comparison, the solely pyrene‐containing nanohoop **65** ([4]CPY) was published by Yamago in the same year.^[120]^ Due to solubility issues, the synthesis started from tetrahydropyrene, which was transformed into the corresponding stannane and cyclized with a platinum complex (Scheme 1 c). Reductive elimination yielded the tetrahydropyrene‐based nanohoop precursor, which was then dehydrogenated with Pd/C to yield **65**. As an [8]CPP analogue, the absorption and emission were both blueshifted by about 30 nm to 311 nm and 500 nm, respectively, with a low FQY of *Φ*_F_=0.05, similar to [8]CPP (Figure 18 d). **65** showed a concentration‐dependent shift of the emission, indicating the formation of pyrene excimers in solution.

#### 6.2.7 Hexabenzocoronene, Tetraphene, and Pentaphene

**Figure 20:**
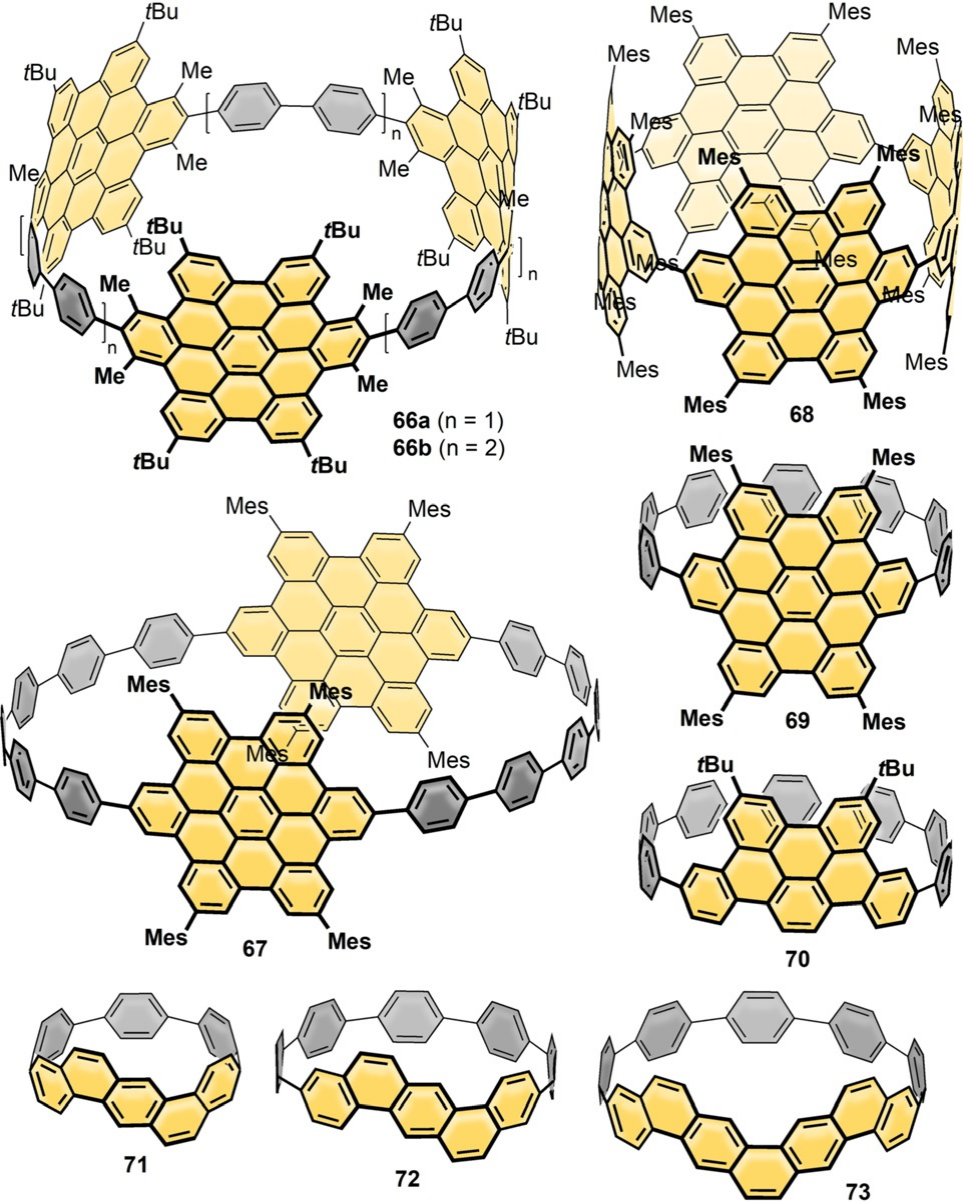
Hexabenzocoronene‐containing nanohoops **66**,^[123]^ **67**,^[125]^ **68**,^[127]^ and **69**,^[128]^ tribenzo[*fj*,*ij*,*rst*]pentaphene‐based hoop **70**,^[128]^ and benzotetraphene‐ (**71** and **72**) and dibenzopentaphene‐ (**73**) containing hoops.^[129]^

As a disc‐shaped subunit of graphene, hexabenzocoronene (HBC) has been used as a starting point to create larger graphenic structures.^[122]^ Incorporating HBC as well as substructures thereof into CPPs provides templates for the synthesis of structurally defined SWNTs (Figure 20). The first HBC‐containing nanohoop **66 b** was reported by Nishiuchi, Müllen, and co‐workers in 2015.^[123]^ Synthesis was conducted using a post‐construction method, where the HBC was formed in a Scholl reaction after the hoop's synthesis. This had been attempted before by the same group, but the cyclodehydrogenation had been hampered by, among others, 1,2‐phenyl shifts.^[124]^ To circumvent this, methyl groups were introduced at the critical positions. The [15]‐ and [21]CPP precursors to **66 a** and **66 b** were synthesized using Jasti corner units (Scheme 1 a) and a Ni‐mediated coupling, followed by successful cyclodehydrogenation for the larger **66 b**. NMR analysis proved a symmetric structure for **66 b**, and the absorption and emission spectra showed patterns distinctive for substituted HBC units.

**Figure 21:**
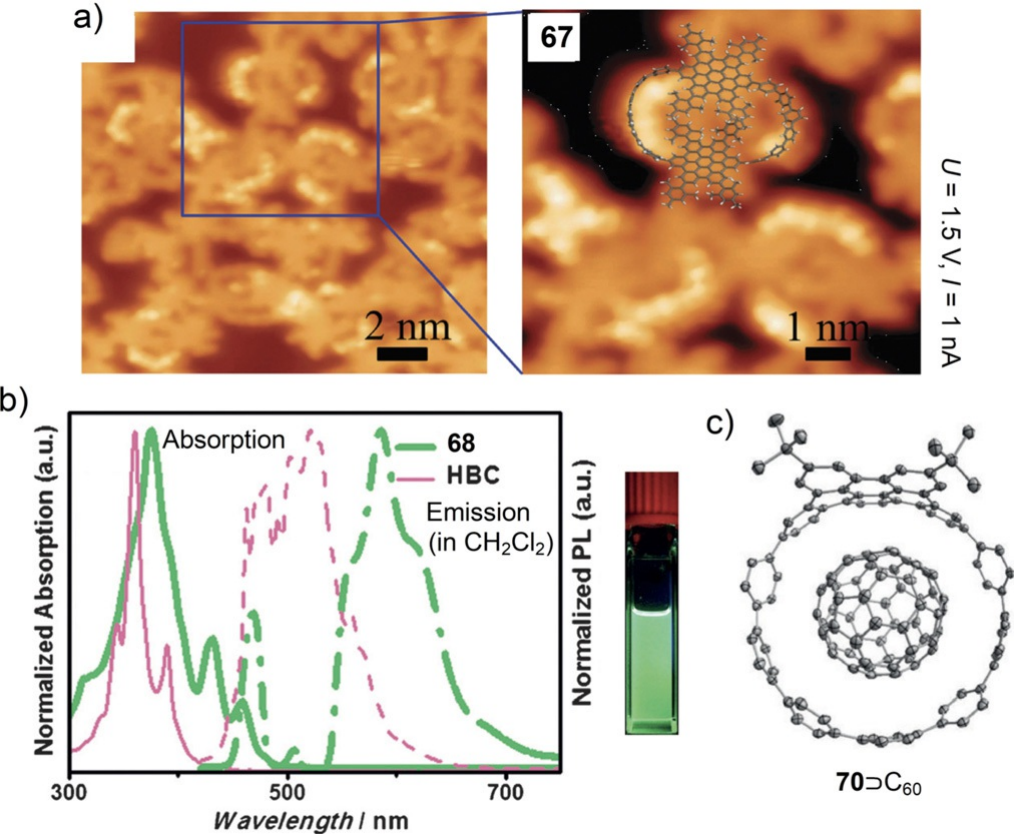
a) STM images of **67** on Au(111) in ultrahigh vacuum, reproduced from ref. [125] with permission from The Royal Society of Chemistry. b) Optical spectra of **68** and HBC and photograph of emission color, reprinted with permission from ref. [127]; copyright 2017 Wiley‐VCH. c) Molecular structure of the C_60_ complex of **70** in the solid state (thermal ellipsoids shown at 50 % probability level, hydrogen atoms omitted for clarity), reprinted with permission from ref. [128]; copyright 2019 Wiley‐VCH.

In 2016 the Du group reported HBC‐containing [18]CPP **67**, which was synthesized using Itami's corner unit (Scheme 1 b) together with bis‐borylated HBC units and a Ni‐mediated ring‐closing reaction.[^125^, ^126^] Its cyclic structure was confirmed by STM on Au(111) (Figure 21 a). With 375, 431, and 458 nm, the optical absorption bands were redshifted compared to both [18]CPP and HBC, the same was observed for the emissions. DFT calculations showed that in both HOMO and LUMO the electron density was localized on the HBC units.

In 2017 the Du group followed up with the cyclic tetramer of tetramesityl HBC **68**, synthesized through the Pt‐mediated coupling (Scheme 1 c) of bis‐borylated HBCs.[^23^, ^126^, ^127^] NMR spectroscopy confirmed the high symmetry. Although it is a more constrained derivative of [12]CPP, with 49 kcal mol^−1^ its strain energy was estimated to be slightly lower than that of the latter (55 kcal mol^−1^). The extended conjugation led to a redshift in both absorption (375, 431, and 458 nm) and emission (468, 506, and 586 nm) compared to both [12]CPP and the HBC reference compound (Figure 21 b). The host–guest chemistry with fullerene‐C_70_ was investigated and provided an association constant of 1.1×10^6^ m ^−1^ (in toluene), which is higher than those of [10]CPP⊃C_70_ (8.4×10^4^ m ^−1^) and [11]CPP⊃C_70_ (1.5×10^5^ m ^−1^, both in toluene), while [12]CPP, with a diameter similar to that of **68**, did not bind C_70_.^[130]^

The most recent and most strained example is the HBC‐containing [10]CPP derivative **69** reported by the Du group in 2019.^[128]^ For the synthesis, Du's C‐shaped synthon for a [7]paraphenylene linker based on three Jasti corner units (Scheme 1 a) was used in a Suzuki–Miyaura reaction with bis‐borylated HBC. Using the same method, tribenzo[*fj*,*ij*,*rst*]pentaphene (TBP)‐based hoop **70** was also synthesized, both hoops in an impressive >1 g quantity. While **70** showed a slight blueshift of its first absorption band (325 nm, second band at 356 nm) compared to [10]CPP (338 nm), the absorptions of the more π‐extended **69** were redshifted (360, 378 and 446 nm). The same behavior was observed for the emissions. A binding study provided a higher association constant with C_60_ for **69** (2.3×10^7^ m ^−1^) than for **70** (3.3×10^6^ m ^−1^) (in toluene), which was larger than that of [10]CPP (2.8×10^8^ m ^−1^) due to the vertical π‐extension of the hoop. X‐ray diffraction showed C_60_ to be centrally located in the hoop of TBP‐based **70** (Figure 21 c).

In 2016, the Jasti group incorporated benzotetraphene and dibenzopentaphene into CPP scaffolds (**71**–**73**) to demonstrate a new concept for the synthesis of conjugated nanobelt fragments.^[129]^ The authors used a combination of CPP synthetic strategies, including the Jasti corner (Scheme 1 a) and a Suzuki–Miyaura coupling for hoop closure, and the olefin metathesis of vinyl side groups to close the second set of six‐membered rings. With 106, 79, and 71 kcal mol^−1^ the strain energies for **71**–**73** were 5–9 kcal mol^−1^ higher than those of the corresponding [6]‐, [8]‐, and [9]CPPs. The major absorptions of **72** at 310 nm and **73** at 325 nm were blueshifted compared to [*n*]CPPs (340 nm), and cyclic voltammetry indicated slightly higher HOMO energies than for [*n*]CPPs.

## 7 Conjugated Nanobelts

We will close this Review with conjugated nanobelts—the synthetically most challenging hoop derivatives. Cyclacenes, conjugated nanobelts consisting of annelated small rings, have been discussed since 1954 in connection with the conjugation in hoop‐shaped π‐systems.[^2^, ^6^] In particular, [6]_*n*_cyclacenes were long‐standing synthetic targets,[^4^, ^5^, ^9^, ^10^] but they could never be synthesized as isolable species due to their predicted open‐shell character.[^10^, ^131^] Recently, [6]_8_cyclacene was detected in the gas phase,^[132]^ providing evidence of its successful synthesis. After acceptance of this manuscript, the first two reports of benzannulated [6]_*n*_cyclacenes, named zigzag carbon nanobelts, appeared, as will be discussed below. Changing to an angular annelation pattern of the six‐membered rings or incorporating ring sizes other than six, on the other hand, successfully led to the synthesis of several conjugated nanobelts to date, as detailed below.[^133^, ^134^] Heterocyclacenes[^135^, ^136^] and nanobelts incorporating pentalene units^[137]^ have also been identified as attractive synthetic targets.

### 7.1 Nanobelts with Annelated Four‐, Six‐, and Eight‐Membered Rings

**Figure 22:**
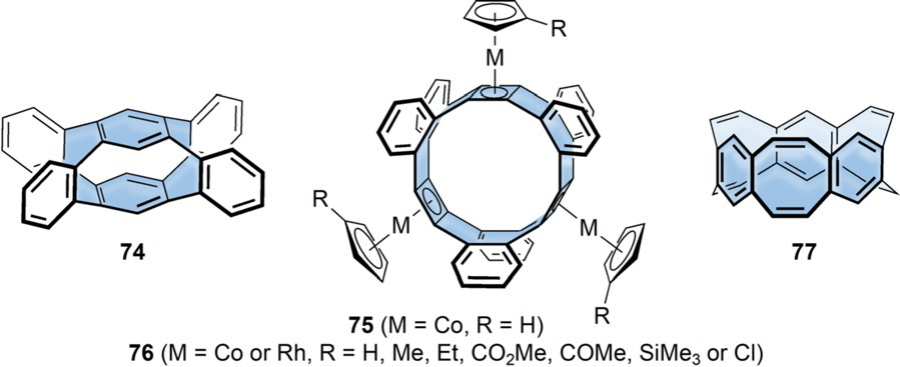
Conjugated nanobelts **74**,^[138]^ **75** and **76**,[^139^, ^140^] and **77**.[^141^, ^142^]

The very first example of a conjugated nanobelt was reported by the Wudl group in 2002 with [6.8]_2_cyclacene **74** (Figure 22).^[138]^ **74** was synthesized by successive [4+2] cycloadditions of dibenzocyclooctadiyne using a reactive pyrimidinium betaine as the diene, and its structure was confirmed by X‐ray diffraction. The absorption spectrum featured two bands at 225 and 285 nm and a broad band at 330 nm, significantly redshifted compared to other cyclophanes. In 2004 the Gleiter group introduced Co‐capped cyclobutadiene rings into nanobelts in the form of [4.8]_3_cyclacenes **75**.^[139]^ The synthesis used the Co‐mediated alkyne dimerization reaction to link three dibenzocyclooctadiynes into a macrocycle, furnishing nanobelt **75** as the main product. Its structure was confirmed by X‐ray diffraction. The synthetic strategy was extended to derivatives **76** including substituents R at the Cp rings or Rh instead of Co in 2009.^[140]^

In 2008 the Gleiter group reported the bottom‐up synthesis of the first unsubstituted conjugated nanobelt, namely [6.8]_3_cyclacene **77**.^[141]^ **77** consists of alternating six‐ and eight‐membered rings and was synthesized by Wittig reactions to make a hexamethyl [2_3_]‐metacyclophane. Oxidation of the methyl to aldehyde groups and a final threefold McMurry coupling reaction for the second set of double bonds led to **77**.^[142]^ X‐ray diffraction proved its *D*_3*h*_‐symmetric structure (Figure 24 a). Its absorption maximum lay at 220 nm with shoulders at 278 and 290 nm, and fluorescence occurred with a maximum at 370 nm. After acceptance of this manuscript, N‐doped [(6.)_*m*_8]_*n*_cyclacenes of different sizes and containing dibenzodiazocines were reported by the Wu group.^[143]^

### 7.2 Nanobelts Solely Consisting of Annelated Six‐Membered Rings

**Figure 23:**
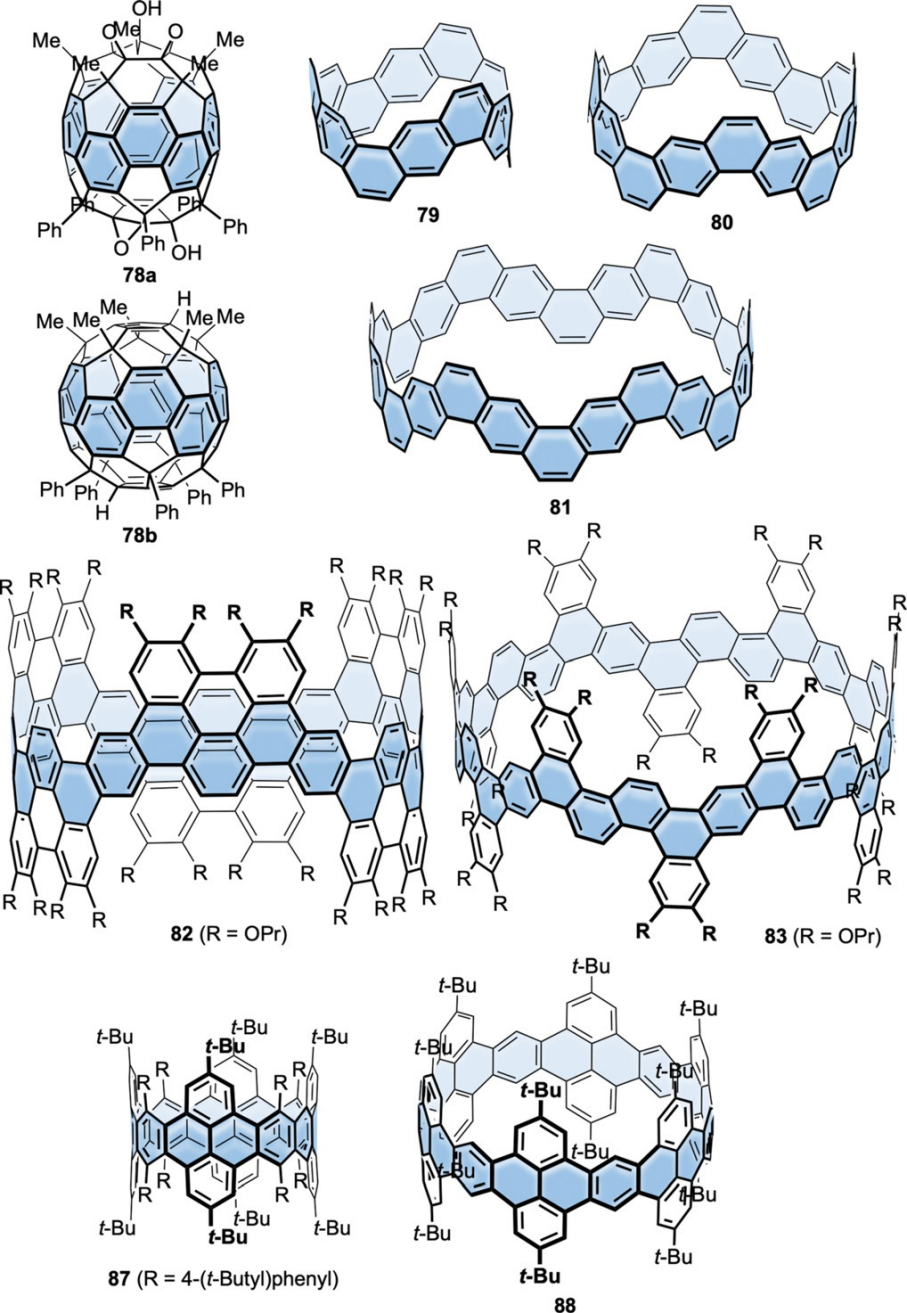
Conjugated nanobelts consisting of annelated six‐membered rings **78**,[^144^, ^145^] **79**,^[146]^ **80** and **81**,^[147]^ **82** and **83**,^[99]^ **87** ^[150]^ and **88**.^[151]^

**Figure 24:**
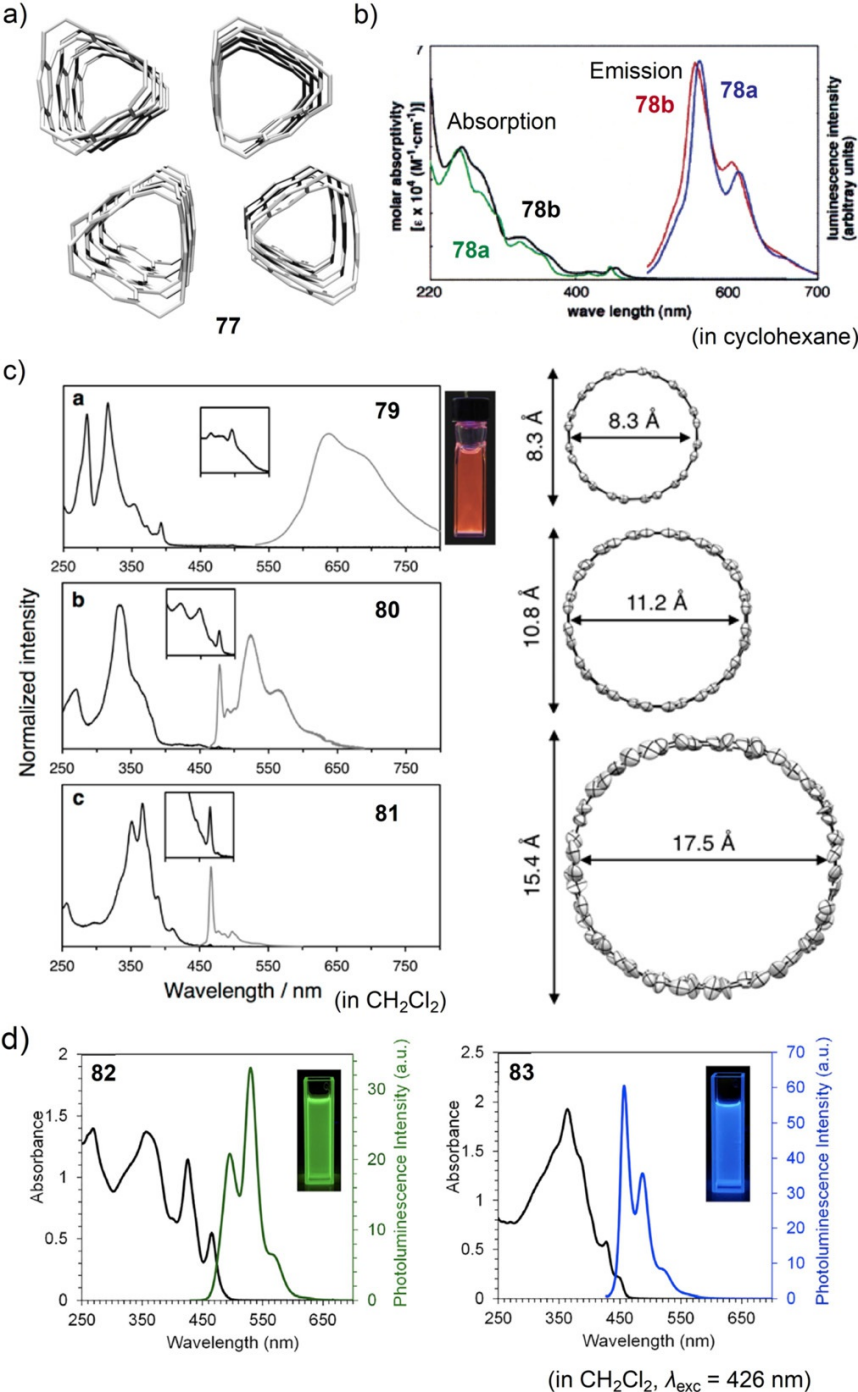
a) Packing of **77** in the solid state, reprinted with permission from ref. [142]; copyright 2009 Wiley‐VCH. b) Optical spectra of **78 a** and **78 b**, reprinted with permission from ref. [145]; copyright 2004 American Chemical Society. c) Optical spectra (with photograph of emission color) and molecular structures in the solid state (thermal ellipsoids shown at 50 % probability level, hydrogen atoms omitted for clarity) of **79**–**81**, from ref. [146] and [147], reprinted with permission from AAAS. https://science.sciencemag.org/content/356/6334/172 and copyright 2018 American Chemical Society. d) Optical spectra of **82** and **83**, reprinted from ref. [99], “Synthesis of Armchair and Chiral Carbon Nanobelts”, copyright 2019, with permission from Elsevier.

In 2003 Nakamura et al. reported on the carbon‐capped [10]cyclophenacene derivatives **78 a** and **b** (Figure 23).[^144^, ^145^] The group approached the challenging synthetic target of a cyclophenacene in a top‐down strategy starting from fullerene‐C_60_ and saturating the top and bottom part using organocopper chemistry. The structure of **78 a** was confirmed by X‐ray diffraction. **78 a** and **b** showed broad absorption bands with maxima at 260 nm and further broad bands that extended up to 500 nm, and they emitted with maxima at 560 and 620 nm (*Φ*_F_=0.10, Figure 24 b).

In 2017, the group of Segawa and Itami was the first to succeed in the bottom‐up synthesis of a carbon nanobelt solely consisting of six‐membered rings, namely **79**, as a [12]cyclophenacene isomer.^[146]^ The synthetic strategy consisted of sequential Wittig reactions to construct a decabrominated [2_5_]‐paracyclophane as a key precursor followed by subsequent Ni‐mediated aryl–aryl coupling. The synthesis was later optimized and **79** made accessible in 0.8 % overall yield from *p*‐xylene.^[147]^ X‐ray diffraction confirmed its *C*_2*h*_‐symmetric structure (Figure 24 c). The ring strain was calculated to be 120 kcal mol^−1^. NICS calculations showed the main resonance form to be the one with most Clar sextets,^[148]^ one in each of the six aryl rings along the equator. **79** displayed several absorption bands reaching up to 550 nm with two maxima at 284 and 313 nm, while the emission was found in the red region with a maximum at 630 nm (Figure 24 c). This seemingly large Stokes shift was due to the fact that the S_0_→S_1_ transition is symmetry‐forbidden, and only the S_0_→S_2_ absorption band was visible. The group recently showed that **79** can undergo sixfold Diels–Alder reaction of its six central six‐membered rings with arynes and alkynes, resulting in cycloiptycenes.^[149]^

In 2018 the Segawa and Itami group published larger congeners **80** and **81** of this nanobelt as [16]‐ and [24]cyclophenacene isomers.^[147]^ Synthesis was performed with the same strategy as for **79** using Wittig reactions and Ni‐mediated couplings. X‐ray diffraction proved the highly symmetric structures of these nanobelts with diameters of 11.2 and 17.5 Å for **80** and **81**, respectively (Figure 24 c). Due to the larger π‐systems, the absorptions were redshifted compared to **79** with maxima at 333 nm (**80**) and 351 and 356 nm (**81**). The emissions, on the other hand, showed interesting features. At room temperature, both the S_1_→S_0_ and S_2_→S_0_ emissions were visible for **80** at 524 and 478 nm (*Φ*_F_=0.13), respectively, while for **81** only the S_1_→S_0_ transition appeared at 466 nm (*Φ*_F_=0.10), as assigned by TDDFT calculations (Figure 24 c).

The Chi and Miao groups reported in 2019 nanobelt **82** as a [24]cyclophenacene isomer as well as chiral **83**.^[99]^ Synthesis was afforded by π‐expansion of the corresponding dodecaaryl‐substituted nanohoops through Scholl reactions starting from a [12]CPP for **82** and a nanohoop consisting of alternating aryl and 2,6‐connected naphthyl units, six of each, for **83**. NMR spectroscopy confirmed the high *D*_3*d*_ and *D*_6_ symmetry with calculated strain energies of 54 and 28 kcal mol^−1^ for **82** and **83**, respectively. Both belts showed broad absorptions up to 500 nm for **82** and 470 nm for **83** with maxima around 350 nm (Figure 24 d). This is similar to the absorption maxima of **81**, which has the same belt size as **82**. The emission spectra also resembled those of **81** with maxima at 498 and 532 nm for **82** and at 464 and 492 nm for **83**, but with higher FQYs (*Φ*_F_=0.35 and 0.46, respectively). Both belts were visualized by STM on a Au(111) surface.

As mentioned above, the very first two reports of zigzag carbon nanobelts appeared in 2021 by Chi^[150]^ (**87**) as well as Segawa and Itami and coworkers (**88**)^[151]^ after acceptance of this manuscript.^[152]^ **87** is a [6]_12_cyclacene derivative, while **88** is a derivative of a [6]_18_cyclacene. In order to overcome the small predicted singlet–triplet gaps of [6]_*n*_cyclacenes, both groups annulated six‐membered rings along the belt rim, resulting in a total of 12 and 18 aromatic sextets, respectively. The syntheses both employed Diels–Alder cycloadditions of furan or furan derivatives with arynes. Noteworthy is the use of hexafluorobenzene as a template in the macrocyclization step during the synthesis of **88**. X‐ray crystallography revealed diameters of 0.92 nm for **87** and 1.4 nm for **88**. In spite of these different sizes, the absorption maxima of **87** at 332 nm with a shoulder peak at 405 nm and of **88** at 336 nm with a small peak at 405 nm were found at similar wavelengths. The emission bands, on the other hand, were shifted to longer wavelength in smaller **87** (peak maxima at 422, 429, and 442 nm) compared to **88** (maxima at 407 and 432 nm).^[152]^

## 8 Conclusions and Outlook

Synthetic advances in the last 13 years in nanohoop synthesis, in particular for the parent [*n*]cycloparaphenylenes, have enabled synthetic chemists to apply these concepts to incorporate a large variety of aromatic groups other than benzene into hoops. As we have shown here, this made it possible to modify the optoelectronic properties of the hoops, that is, by introducing donor or acceptor moieties or even donor–acceptor structures, to change their structural properties and induce e.g. chirality, leading to intriguing chiroptical properties, and to provide vertically extended hoops as model systems and templates for single‐walled carbon nanotubes. These examples provide a base to—in the future—study applications of conjugated nanohoops^[16]^ with designed properties in materials science or biology, to make use of the chiroptical properties of nanohoops in optoelectronic devices, and to further develop methods for the synthesis of single‐chirality carbon nanotubes starting from suitable templates.

## Conflict of interest

The authors declare no conflict of interest.

## Biographical Information

*Mathias Hermann studied chemistry at the University of Freiburg (Germany) and received his B.Sc. degree under the supervision of Prof. B. Breit. He obtained his M.Sc. degree in 2016 under the guidance of Prof. B. Esser and has been a Ph.D. student in her group since then. His research focuses on the synthesis and properties of dibenzopentalenes and dibenzopentalene‐containing nanohoops*.

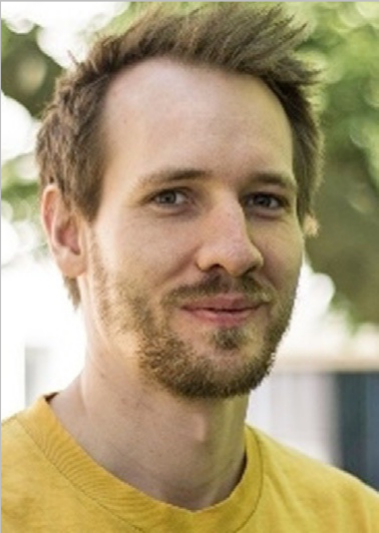

## Biographical Information

*Daniel Wassy received his B.Sc. (2014) and M.Sc. (2016) in Chemistry from the University of Bonn (Germany). Since November 2016 he has been pursuing doctoral studies at the University of Freiburg under the supervision of Prof. B. Esser. His research interests include the synthesis and theoretical investigations of curved molecules incorporating the dibenzopentalene motif*.

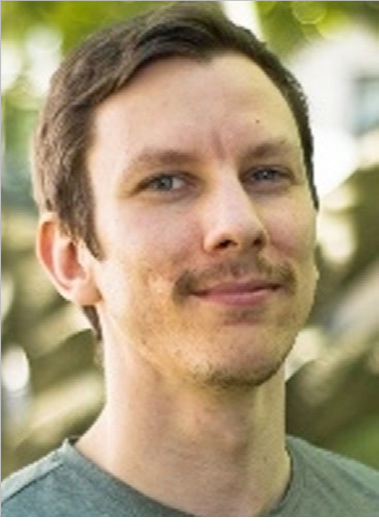

## Biographical Information

*Birgit Esser has been associate professor for Molecular/Organic Functional Materials at the University of Freiburg since 2015. She obtained her Ph.D. in 2008 at the University of Heidelberg. After postdoctoral studies at the Massachusetts Institute of Technology as a Leopoldina fellow, she returned to Germany, to the University of Bonn, where she was an Emmy‐Noether junior group leader from 2012 to 2015. Her research focuses on hoop‐shaped, conjugated π‐systems, small‐molecule semiconductors, and organic electrode materials for batteries*.

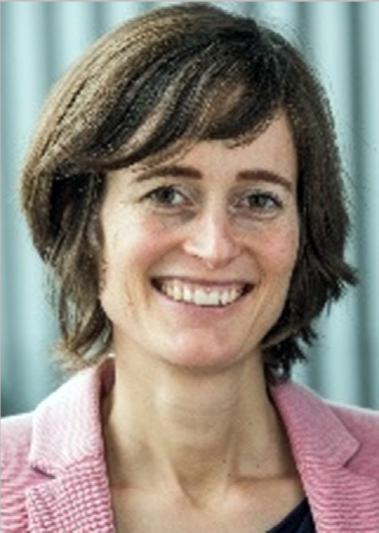

## Supporting information

As a service to our authors and readers, this journal provides supporting information supplied by the authors. Such materials are peer reviewed and may be re‐organized for online delivery, but are not copy‐edited or typeset. Technical support issues arising from supporting information (other than missing files) should be addressed to the authors.

Supplementary
