# Supplementary material for: Conjugated Nanohoops Incorporating Donor, Acceptor, Hetero‐ or Polycyclic Aromatics
Source: Angew Chem Int Ed Engl. 2021 Mar 22;60(29):15743–66. doi: 10.1002/anie.202007024 (PMC9542246; doi:10.1002/anie.202007024)
Supplement: Supplementary file 1 — Supplementary [file ANIE-60-15743-s001.pdf]

Angewandte Chemie
Eine Zeitschrift der Gesellschaft Deutscher Chemiker

Supporting Information

Conjugated Nanohoops Incorporating Donor, Acceptor, Hetero- or Polycyclic Aromatics

*Mathias Hermann[+], Daniel Wassy[+], and Birgit Esser**

anie_202007024_sm_miscellaneous_information.pdf

Table S1. Optoelectronic properties of nanohoops and nanobelts from chapters 6 and 7.

Compound	λ_{max}[nm]	ε [M^{-1}cm^{-1}]	λ_{em}[nm]	Φ_F	$E_{1/2,Ox}$ [V][a]	$E_{1/2,Red}$ [V] [a]	E_{HOMO}[eV][b]	E_{LUMO}[eV][b]	E_{gap}[eV][b]
**[3]40a**	355 ($CHCl_3$)	35000	-	-	0.21[c], 0.40[c]	−1.77[d]	−4.66	−1.78	2.88
**[3]40a**	342 ($CHCl_3$)	24000	-	-	0.17[c], 0.38[c]	−1.71[d]	−4.55	−1.89	2.66
**[4]40a**	350 ($CHCl_3$)	64000	514 ($CHCl_3$)	0.32	0.45[c]	-	−4.90	−1.61	3.29
**[4]40b**	347 (CyH)<br>341 (THF)<br>342 ($CHCl_3$)<br>340 (MeCN)	90000 (CyH)	487 (CyH)<br>491 (THF)<br>492 ($CHCl_3$)<br>493 (MeCN)	0.25<br>(CyH)	1.02[c, k, l]	−2.23[c, j, l]	−5.26[h]	−2.34[i]	2.92
**[4]40c**	349 (THF)	-	512 (THF)	0.45	0.61[c]	-	−4.89	−1.61	3.28
**[4]40d**	349 (CyH)	-	515 (CyH)	0.21	0.88, 0.98[c, k, l]	-	−5.1[h]	−2.2[i]	2.9
**[5]40d**	355 (CyH)	-	440, 466 (CyH)	0.61	1.03, 1.21,<br>1.43[c, k, l]	-	−5.3[h]	−2.8[i]	2.5
**[5]40e**	355 (CyH)	-	443, 466 (CyH)	0.61	1.00, 1.40,<br>1.63, 1.78[c, k, l]	-	−5.3[h]	−2.1[i]	3.2
**[6,6]41**	359 (CH_2Cl_2)	176000	493 (CH_2Cl_2)	0.37	0.63[d]	−2.34[c]	-	-	-
**[8,8]41**	355 (CH_2Cl_2)	255000	468 (CH_2Cl_2)	0.64	0.71[d]	−2.48[c]	-	-	-
**[10,10]41**	353 (CH_2Cl_2)	285000	454 (CH_2Cl_2)	0.86	0.77[d]	−2.51[c]	-	-	-
**43a**	346 (CH_2Cl_2)	229000	450 (CH_2Cl_2)	-	0.72[c], 0.99[c]	−1.87[c]	−4.70[e]	−2.98[e]	1.72[e]
**43b**	342 (CH_2Cl_2)	173000	-	-	0.89[c]	−1.41[c], −1.77[c]	-	-	-
**[8]46**	360	-	-	-	-	-	-	-	-
**47**	390	-	-	-	-	-	-	-	-
**[9]48**	378 ($CHCl_3$)	68000	491 ($CHCl_3$)	0.35	-	-	−4.85	−1.76	3.09
**49**	305 (DMSO)	31600	480 (DMSO)	0.29	-	-	-	-	-
**50a**	362 (CH_2Cl_2)	52000	512 (CH_2Cl_2)	-	-	-	-	-	-
**50b**	346 (DMSO)	19000	445 (DMSO)	-	-	-	-	-	-
**51a**	358 (CH_2Cl_2)	-	483 (CH_2Cl_2)	-	-	-	-	-	-
**51b**	358 (CH_2Cl_2)	-	484 (CH_2Cl_2)	-	-	-	-	-	-
**52**	336 (CH_2Cl_2)	-	453 (CH_2Cl_2)	-	-	-	-	-	-
**59**	338 (CH_2Cl_2)	-	487 (CH_2Cl_2)		0.65[c]		−4.97	−1.91	3.06
**60**	329 (CH_2Cl_2)	-	441 (CH_2Cl_2)	0.68	-	-	−5.08	−1.92	3.16
**62**	320 (CH_2Cl_2)	17000	485 (CH_2Cl_2)	0.47	0.53[c]	-	-	-	-
**63**	276 (CH_2Cl_2)	37000	514 (CH_2Cl_2)	0.18	-	-	−5.11[f]	−2.43[f]	2.68[f]
**64**	343 (CH_2Cl_2)	610000	430 (CH_2Cl_2)	0.21			−5.34	−1.62	3.72
**65**	311 ($CHCl_3$)	17000	500 ($CHCl_3$)	0.05	0.83[d]	−1.77[c]	−5.18	−1.70	3.48
**66b**	367 (CH_2Cl_2)	-	479	-	-	-	−5.08	−1.91	3.17
**67**	371 (CH_2Cl_2)	10000	534 (CH_2Cl_2)	0.08	-	-	−5.10	−1.88	3.22
**68**	375 (CH_2Cl_2)	510000	586 (CH_2Cl_2)	0.08	-	-	-	-	-
**69**	360 (CH_2Cl_2)	70000	481 (CH_2Cl_2)	0.03	-	-	−5.32[g]	−2.12[g]	3.21[g]
**70**	356 (CH_2Cl_2)	87000	459 (CH_2Cl_2)	0.13	-	-	−5.33[g]	−2.08[g]	3.24[g]
**71**	450 (THF)	-	-	-	0.4[c]	−2.15[c]	−4.86	−1.90	2.96
**72**	417 (CH_2Cl_2)	-	535 (CH_2Cl_2)	0.14	0.6[c]	−2.3[c]	−5.06	−1.80	3.26
**73**	409 (CH_2Cl_2)	-	500 (CH_2Cl_2)	0.15	0.75[c]	−2.34[c]	−5.12	−1.80	3.32
**77**	220	49000	370	-	-	-	-	-	-
**78a**	260 (CyH)	-	560	0.10	-	-	-	-	-
**78b**	260 (CyH)	-	560	0.10	-	-	-	-	-
**79**	313(CH_2Cl_2)	148000	630 (CH_2Cl_2)	0.03	-	-	−4.92	−1.97	2.95
**80**	-	-	complex spectrum	0.13	-	-	−5.07	−1.95	3.12
**81**	-	-	466 (CH_2Cl_2)	0.10	-	-	−5.09	−1.94	3.15
**82**	350 (CH_2Cl_2)	-	532 (CH_2Cl_2)	0.35	0.59[c]	-	-	-	-
**83**	364 (CH_2Cl_2)	-	462 (CH2Cl2)	0.46	0.94[d]	-	-	-	-
**87**	347, 370 (CH_2Cl_2)	52480, 22390	-	-	0.03[c], 0.19[c]	-	−4.40	−1.74	2.66
**88**	340 (CH_2Cl_2)	34100	488 (CH_2Cl_2)	0.49	-	-	-	-	-

[a] from cyclic voltammetry *vs.* Fc/Fc$^+$; [b] if not stated otherwise B3LYP/6-31G*; [c] reversible; [d] irreversible; [e] B3LYP/def2-TZVP; [f] CAM-B3LYP/def2-TZVPD; [g] B3LYP/6-31G(d,p); [h] calculated from the onset of the first oxidation in the CV; [i] calculated from the onset of the first reduction in the CV; [j] cathodic peak potential; [k] anodic peak potential; [l] *vs.* SCE.
